# Globally reduced *N*^6^-methyladenosine (m^6^A) in C9ORF72-ALS/FTD dysregulates RNA metabolism and contributes to neurodegeneration

**DOI:** 10.1038/s41593-023-01374-9

**Published:** 2023-06-26

**Authors:** Yini Li, Xiaoyang Dou, Jun Liu, Yu Xiao, Zhe Zhang, Lindsey Hayes, Rong Wu, Xiujuan Fu, Yingzhi Ye, Bing Yang, Lyle W. Ostrow, Chuan He, Shuying Sun

**Affiliations:** 1 Department of Physiology, Johns Hopkins University School of Medicine, Baltimore, MD 21205, USA.; 2 Brain Science Institute, Johns Hopkins University School of Medicine, Baltimore, MD 21205, USA.; 3 Department of Chemistry and Institute for Biophysical Dynamics, The University of Chicago, Chicago, IL 60637, USA.; 4 Howard Hughes Medical Institute, Chicago, IL 60637, USA.; 5 Present: State Key Laboratory of Protein and Plant Gene Research, School of Life Sciences, Peking-Tsinghua Center for Life Sciences, Peking University, Beijing 100871, China.; 6 Department of Neurology, Johns Hopkins University School of Medicine, Baltimore, MD 21205, USA.; 7 Cellular and Molecular Physiology Graduate Program, Johns Hopkins University School of Medicine, Baltimore, MD 21205, USA.; 8 Laboratory of Cellular and Developmental Biology, NIDDK Intramural Research Program, Bethesda, MD 20892, USA.; 9 Department of Neurology, Lewis Katz School of Medicine at Temple University, Philadelphia, PA 19122, USA; 10 Department of Biochemistry and Molecular Biology, The University of Chicago, Chicago, IL 60637, USA.; 11 The Solomon H. Snyder Department of Neuroscience, Johns Hopkins University School of Medicine, Baltimore, MD 21205, USA.; 12 Department of Pathology, Johns Hopkins University School of Medicine, Baltimore, MD 21205, USA.

## Abstract

Repeat expansion in *C9ORF72* is the most common genetic cause of amyotrophic lateral sclerosis (ALS) and frontotemporal degeneration (FTD). Here, we show that *N*^6^-methyladenosine (m^6^A), the most prevalent internal mRNA modification, is downregulated in C9ORF72-ALS/FTD patient-derived iPSC-differentiated neurons and postmortem brain tissues. The global m^6^A hypomethylation leads to transcriptome-wide mRNA stabilization and upregulated gene expression, particularly for genes involved in synaptic activity and neuronal function. Moreover, the m^6^A modification in the *C9ORF72* intron sequence upstream of the expanded repeats enhances RNA decay via the nuclear reader YTHDC1, and the antisense RNA repeats can also be regulated through m^6^A modification. The m^6^A reduction increases the accumulation of repeat RNAs and the encoded poly-dipeptides, contributing to disease pathogenesis. We further demonstrated that by elevating m^6^A methylation we could significantly reduce repeat RNA levels from both strands and the derived poly-dipeptides, rescue global mRNA homeostasis, and improve survival of C9ORF72-ALS/FTD patient iPSC-derived neurons.

## Introduction

Dysfunction of RNA metabolism has emerged as key player in multiple neurological disorders^1^. Amyotrophic lateral sclerosis (ALS), a disease of motor neuron degeneration and motor symptoms, is increasingly recognized to have clinical, pathological and genetic overlaps with frontotemporal dementia (FTD), a neurodegenerative disease characterized by behavioral and language dysfunction^2^. Pathological inclusions and/or causative genetic mutations in several RNA-binding proteins (RBP) are widely found in the two diseases, such as TDP-43, FUS/TLS, ATXN2, MATR3 and hnRNP family proteins^3^. Alternatively, the RNA-mediated toxicity could also arise from microsatellite repeat expansions^2^. The most common genetic cause of both ALS and FTD is the hexanucleotide GGGGCC repeat expansion in the first intron of the *C9ORF72* gene^2^. The repeat-containing RNAs form nuclear granules that could disturb RBP functions, and encode multiple dipeptide repeat (DPR) proteins with various toxicities^4^. Therefore, it is important to understand how the dysregulation of RNA metabolisms contributes to disease mechanisms.

In previous efforts to identify genetic modifiers of DPR production via a genome-wide CRISPR-Cas9 knockout screen, we found core components of the m^6^A writer complex, METTL3, and METTL14, as suppressors of DPR production^5^. *N*^6^-methyladenosine (m^6^A) is the most prevalent internal modification in eukaryotic mRNA^6,7^. It is one of the few reversible RNA modifications that regulates RNA metabolism. m^6^A is installed by the “writer” methyltransferase complex composed of core subunits METTL3 and METTL14^8^, and can be removed by the “eraser” protein, FTO or ALKBH5 demethylase^9-11^. Proteins that selectively recognize m^6^A-marked RNA, namely “readers”, showed distinct and overlapping functions in modulating multiple steps of RNA processing, including splicing, degradation and translation, which is recognized as epitranscriptomic regulation^9,12-15^. It is noted that m^6^A in the nervous system showed the highest abundance^7^ and strongest specificity^16^ compared to other tissues. Disruption of the components of m^6^A pathway impaired the development and function of the nervous system^17^. These indicate the importance of the m^6^A-mediated epitranscriptome in preserving neuronal integrity.

Here, we discovered that m^6^A RNA modification is abnormally reduced in C9ORF72-ALS/FTD. This m^6^A hypomethylation leads to transcriptome-wide gene expression dysregulation and increases the accumulation of both sense and antisense repeat RNAs and DPRs. Restoration of the m^6^A level by modulating either methyltransferase or demethylase can rescue the disease-related phenotypes. Our findings reveal a novel layer of RNA regulation that plays a critical role in the pathogenic mechanism of neurodegeneration.

## Results

### The m^6^A RNA modification is downregulated in C9ORF72-ALS/FTD

**Extended Data Fig. 1:**
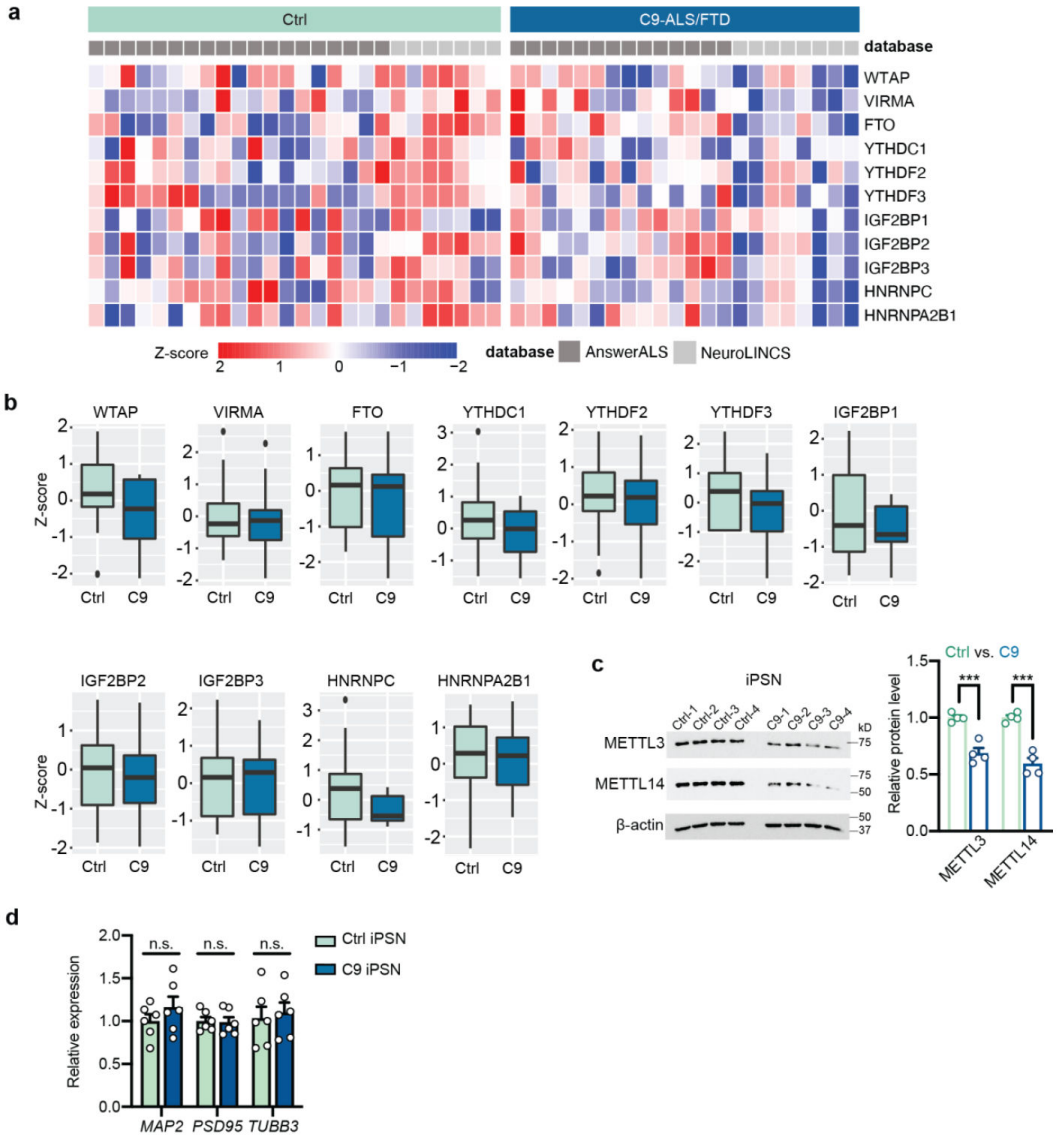
m^6^A pathway-related gene expression in C9ORF72-ALS/FTD iPSNs. **a**, Proteomic heatmap of the m^6^A pathway components in control (n=26) and C9ORF72-ALS/FTD (n=22) iPSNs. Data were normalized to the batch controls. **b**, Boxplots of the proteomic data in (**a**). Box plots indicate the interquartile range with the central line representing the median, and the vertical lines extend to the extreme values in the group. **c**, Western blotting (left) and quantification (right) of METTL3 and METTL14 expression in control and C9ORF72 iPSNs. Data are mean ± s.e.m. ***p=0.0008 and 0.0003, respectively, by two-tailed *t* test. **d**, Relative RNA expression of neuronal marker genes (*MAP2, PSD95* and *TUBB3*) was measured by RT-qPCR in control and C9 iPSNs, showing comparable neuron maturity between control and C9. Points represent individual control or patient lines. n=6 in each group. Data are mean ± s.e.m. MAP2: *p*=0.304; PSD95: *p*=0.86; TUBB3: *p*=0.716, by two-tailed *t* test.

**Extended Data Fig. 2:**
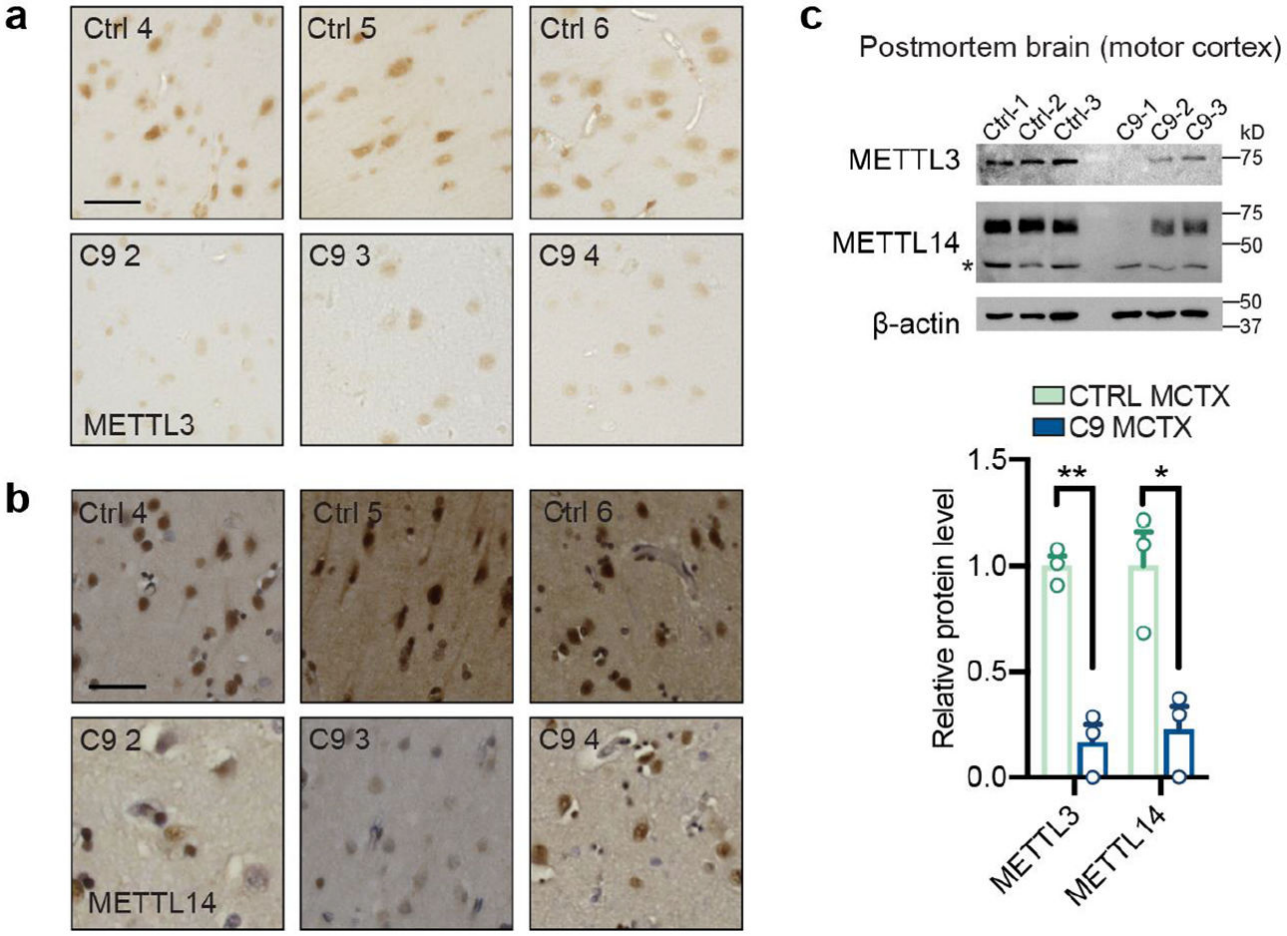
METTL3 and METTL14 m^6^A methyltransferases were reduced in C9ORF72-ALS/FTD postmortem brain. **a**,**b**, Immunohistochemistry (IHC) staining of METTL3 (**a**), METTL14 (**b**) at temporal cortex section of control and C9ORF72-ALS/FTD samples. Scale bar = 50μm. **c**, Western blotting (top) and quantification (bottom) of METTL3, METTL14 using postmortem motor cortex tissue samples. *Non-specific bands. Points represent individual control or patient samples. n=3 in each group. Data are mean ± s.e.m. *p=0.0171, **p=0.0011, by two-tailed *t* test.

**Extended Data Fig. 3:**
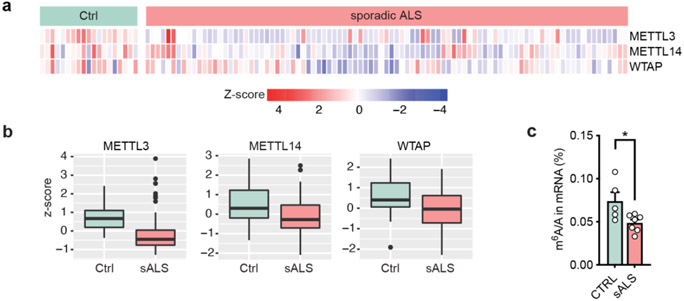
Expression changes of the m^6^A “writer” core components in sporadic ALS iPSNs. **a**, Proteomic heatmap of the core components of m6A “writer” complex in control (n=19) and sporadic ALS (n=93) iPSNs. Data were normalized to the batch controls. **b**, Boxplots of the proteomic data in (**a**). Box plots indicate the interquartile range with the central line representing the median, and the vertical lines extend to the extreme values in the group. **c**, UHPLC-QQQ-MS/MS quantification of the m^6^A/A ratio in poly-A mRNAs from control (n=5) and sporadic ALS patient (n=7) postmortem frontal cortex. Data are mean ± s.e.m. Points represent individual samples. *p=0.0247 by two-tailed *t* test.

**Fig. 1:**
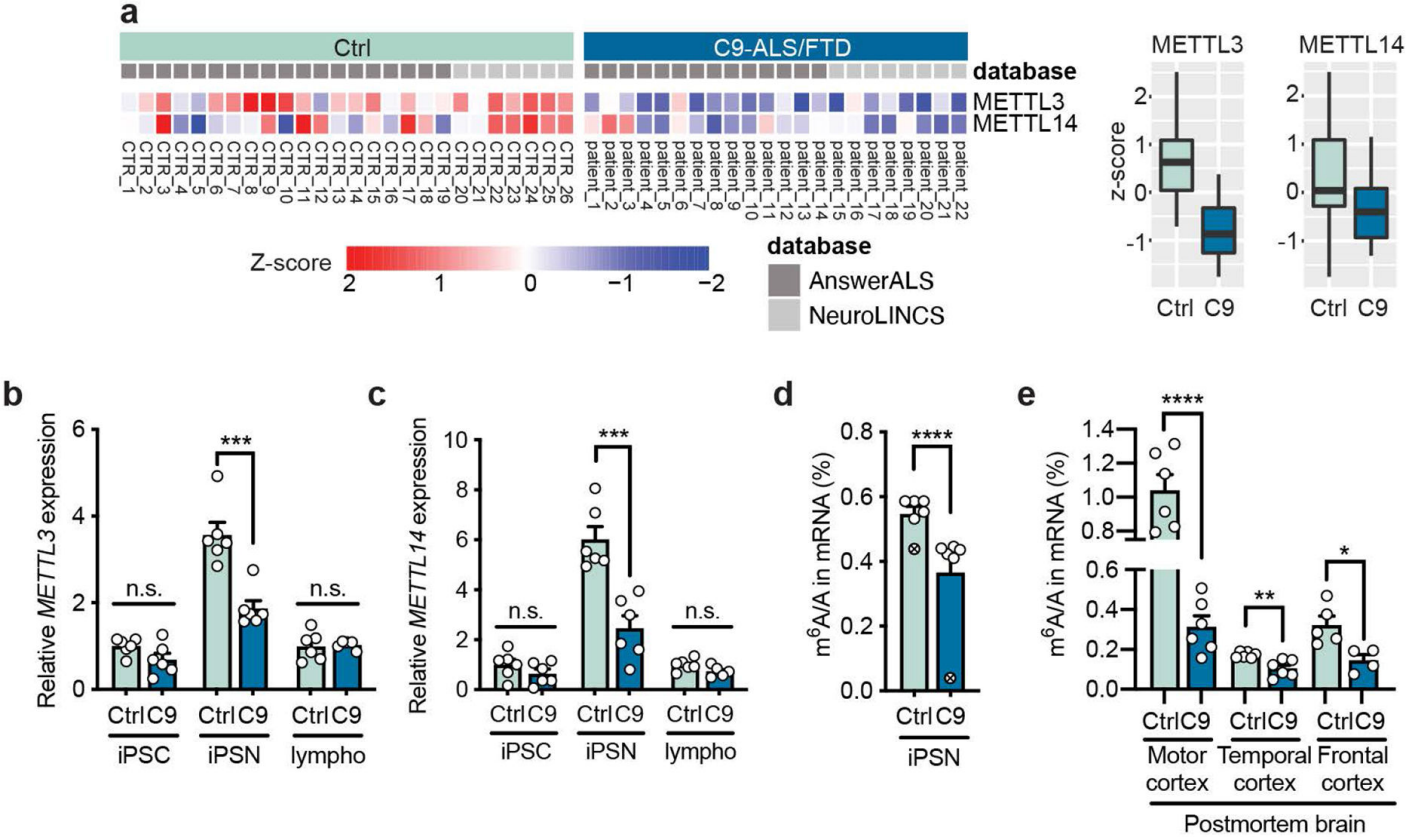
The expression of m^6^A methyltransferases and the m^6^A RNA modification levels are downregulated in C9ORF72-ALS/FTD. **a**, Proteomic heatmap (left) and boxplots (right) of the m^6^A “writer” complex core components in the iPSNs of control (n=26) and C9ORF72-ALS/FTD patients (n=22). Data were analyzed using the proteomic database from Answer ALS and NeuroLINC center. Batch controls were used for normalization of the AnswerALS data. Box plots indicate the interquartile range with the central line representing the median, and the vertical lines extend to the extreme values in the group. **b**,**c**, *METTL3*(**b**) and (**c**) *METTL14* RNA expression levels were measured by RT-qPCR in control and C9 iPSC, iPSN and lymphoblast cells. Points represent individual cell lines, including one pair with isogenic control in which the repeat expansion was removed^71^. The isogenic control iPSC was set to 1. n=6 for both control and patient iPSC lines. n=6 for control and 5 for C9ORF72-ALS lymphoblast lines. Date are mean ± s.e.m. *METTL3*: iPSC, *p*=0.1042; iPSN, ***p=0.0006; lympho, *p*=0.8356. *METTL14*: iPSC, *p*=0.2328; iPSN, ***p=0.0006; lympho, *p*=0.0944. Two-tailed *t* test. **d**, UHPLC-QQQ-MS/MS quantification of the m^6^A/A ratio in mRNAs from iPSNs. The mRNAs of the isogenic pair (circle-cross) were extracted by poly-A selection; the mRNAs of the other iPSN lines were extracted by ribosomal RNA depletion. Two-tailed *t* test was performed on 5 pairs of control and C9 lines (ribosomal RNA depletion samples). Date are mean ± s.e.m. ****p<0.0001 by two-tailed *t* test. **e**, UHPLC-QQQ-MS/MS quantification of the m^6^A/A ratio in mRNAs from postmortem brain tissues extracted by poly-A selection. Points represent individual patient samples. n=6 for both control and patient samples. Date are mean ± s.e.m. *p=0.0168, **p=0.0039, ****p<0.0001, by two-tailed *t* test.

We analyzed the public proteomic data from Answer ALS and NeuroLINC, and found that the expression levels of m^6^A pathway components, especially the methyltransferases METTL3 and METTL14 in the “writer” complex, were markedly downregulated in C9ORF72-ALS/FTD patient-derived induced pluripotent stem cell (iPSC)-differentiated spinal neurons (iPSNs) (Fig. 1a and Extended Data Fig. 1a,b). Consistently, we also observed reduction of METTL3 and METTL14 in multiple additional patient iPSN lines in our lab (Fig. 1b,c and Extended Data Fig. 1c). We confirmed the difference was not due to the variation of neuron maturation or purity between control and patient lines (Extended Data Fig. 1d). Interestingly, no expression changes of *METTL3/14* were observed in patient iPSCs and lymphoblast cells (Fig. 1b,c), suggesting probable neuron-specific downregulation of the m^6^A methyltransferase components. Furthermore, the METTL3 and METTL14 levels also decreased in C9ORF72-ALS/FTD patient postmortem brain tissues (Extended Data Fig. 2a-c). We further quantified the m^6^A/A ratio in purified mRNAs using the ultra-high-performance liquid chromatography-tandem mass spectrometry (UHPLC-QQQ-MS/MS) method^18^. The m^6^A level was dramatically downregulated in both C9ORF72-ALS/FTD iPSNs and multiple regions of patient postmortem brain (Fig. 1d,e). Altogether, this indicates the profound dysregulation of m^6^A pathway in C9ORF72-ALS/FTD patients, which could likely perturb mRNA metabolism globally. Interestingly, the proteomic data suggested iPSNs of sporadic ALS also have reduced expression of the m^6^A writer components in comparison to the controls (Extended Data Fig. 3a,b), and the m^6^A abundance was found to be reduced in sporadic ALS patient postmortem tissues (Extended Data Fig. 3c). We focused on the C9ORF72-ALS/FTD in this work, which could shed light on future studies of sporadic diseases.

### The repeat RNA downregulates METTL3 and METTL14 expression

**Extended Data Fig. 4:**
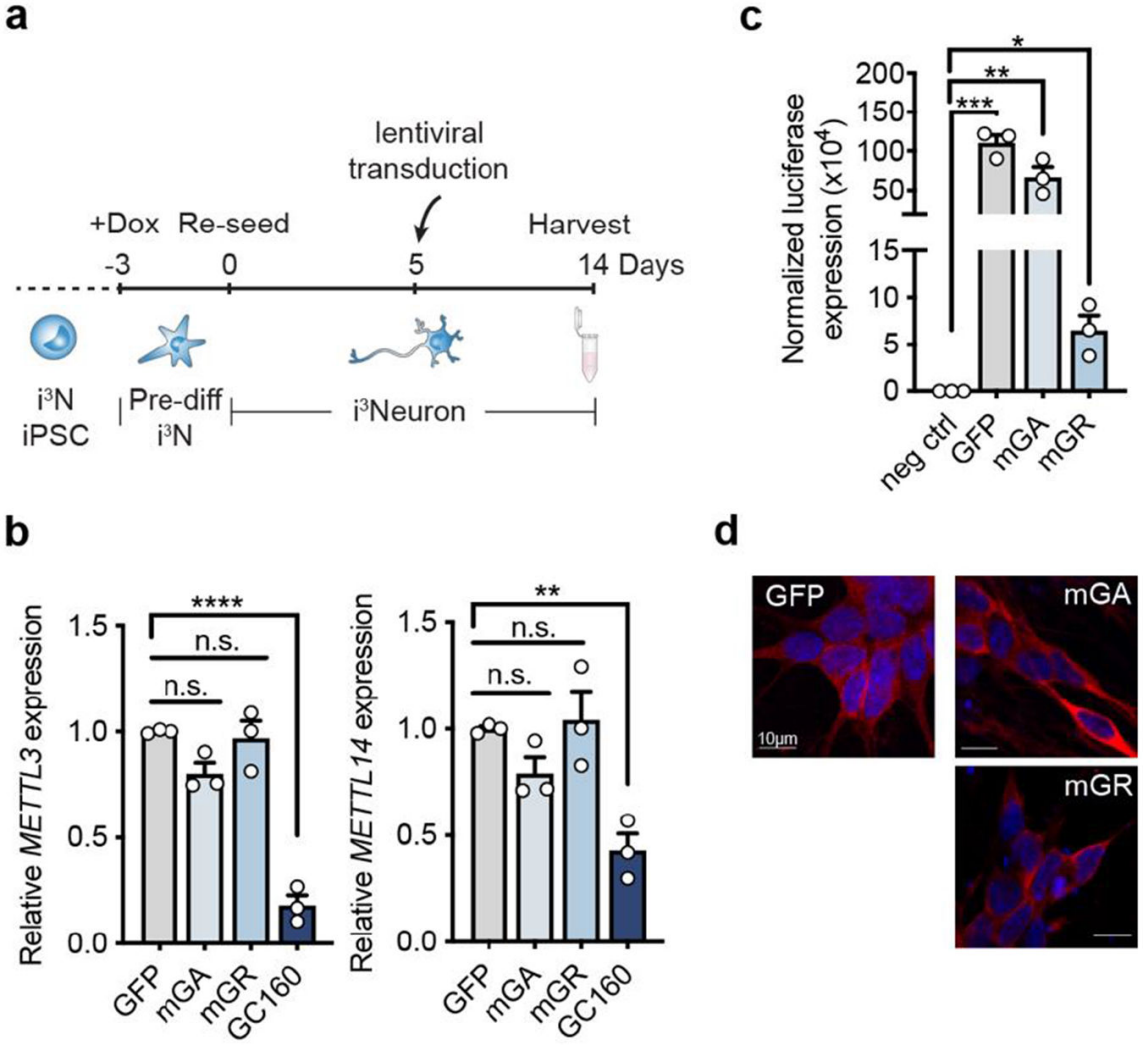
The repeat RNA induces downregulation of METTL3 and METTL14 expression. **a**, Timeline for doxycycline-induced neuron differentiation, lentiviral transduction of transgenes to express repeats or DPRs, and functional analysis of i^3^Neurons. **b**, Relative RNA expression of *METTL3* and *METTL14* in i^3^Neurons expressing GFP control, modified poly-GA, modified poly-GR (with randomized codons), or (GGGGCC)_160_ repeats. *METTL3*: mGA, *p*=0.1154; mGR, *p*=0.9755; GC160, ****p<0.0001. *METTL14*: mGA, *p*=0.3892; mGR, *p*=0.9853; GC160/GFP, **P=0.0073. p-values were calculated by one-way analysis of variance (ANOVA) with Tukey’s multiple comparisons **c**, Detection of GFP, modified poly-GA and modified poly-GR post lentiviral transduction in i^3^Neurons by Nano-Glo HiBiT bioluminescence measurement. The luciferase signal was normalized to the total protein level. i^3^Neurons without lentiviral infection served as the negative control. *p=0.0148, **p=0.0062, ***p=0.0004, by two-tailed *t* test. **d**, Immunofluorescent staining of GFP, modified poly-GA or modified poly-GR in transduced i^3^Neurons. Neurons were stained using FLAG antibody against the C-terminal FLAG epitope tag in each construct. (**b,c**) Points represent three biological replicates. Data are mean ± s.e.m.

We examined whether the C9ORF72 repeat expansion can affect the METLL3 and METTL14 expression level via gain of toxicity. As the m^6^A reduction was only observed in differentiated iPSNs, we performed the experiments in the human i^3^Neuron system, in which the engineered human iPSC line contains doxycycline-inducible neurogenin-2 (NGN2) and can be differentiated into cortical neurons with high efficiency and homogeneity^19^. We expressed the 160× GGGGCC repeats or 50× GA or GR poly-dipeptides encoded by randomized codons in the differentiated i^3^Neurons via lentiviral transduction, and examined the endogenous *METTL3* and *METTL14* levels (Extended Data Fig. 4a). We found that the expression of GGGGCC repeats was sufficient to induce the reduction of the two methyltransferases (Extended Data Fig. 4b). The expression of DPRs from randomized codons did not have significant effect (Extended Data Fig. 4b), even though they were abundantly expressed in the neurons, confirmed by both bioluminescent assay of the N-terminal HiBiT tag and immunofluorescence of the C-terminal FLAG tag of GFP and DPRs (Extended Data Fig. 4c,d). It is noted that poly-GR had lower expression level than poly-GA in the i^3^Neurons (Extended Data Fig. 4c), which is in agreement with the observation in patients^20,21^. Overall, this result indicates that the repeat RNA triggers the m^6^A dysregulation in C9ORF72 patient neurons.

### Transcriptome-wide m^6^A hypomethylation in C9ORF72-ALS/FTD

**Extended Data Fig. 5:**
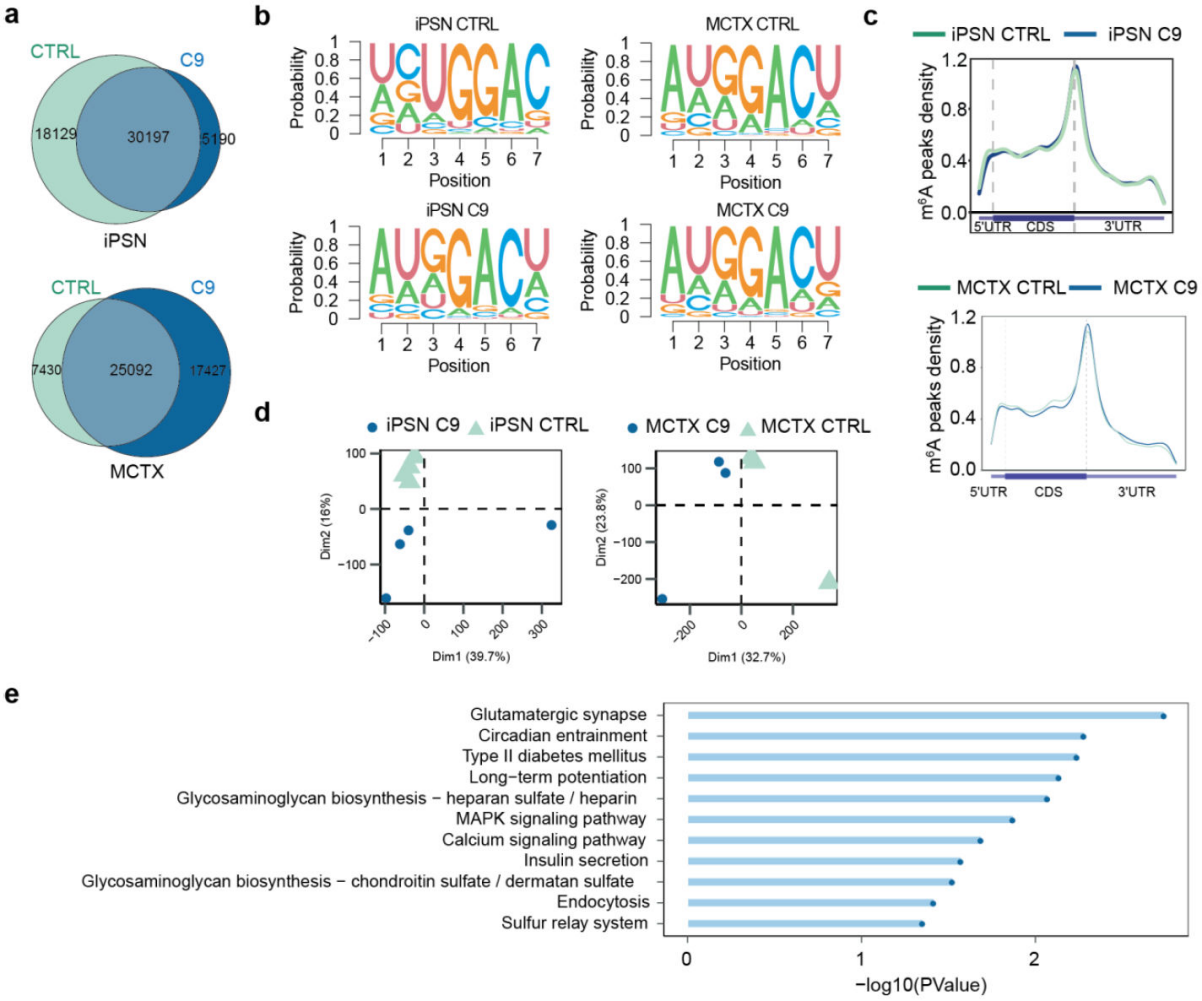
MeRIP-seq in control and C9ORF72-ALS/FTD iPSNs and postmortem motor cortex. **a**, Venn diagram depicting the total number and overlaps of m^6^A peaks detected in control and C9ORF72-ALS/FTD groups of iPSN (top) and motor cortex (bottom). **b**, The most enriched consensus m^6^A motifs in all conditions. **c**, Metagene profiles of m^6^A peak density along transcripts with three non-overlapping segments (5’ UTR, CDS, and 3’ UTR) for control and C9ORF72-ALS/FTD groups. **d**, Principal component analysis (PCA) of control and C9ORF72-ALS/FTD samples. **e**, Gene Ontology (GO) enrichment analysis of hypomethylated genes that were identified in both iPSNs and motor cortex.

**Fig. 2:**
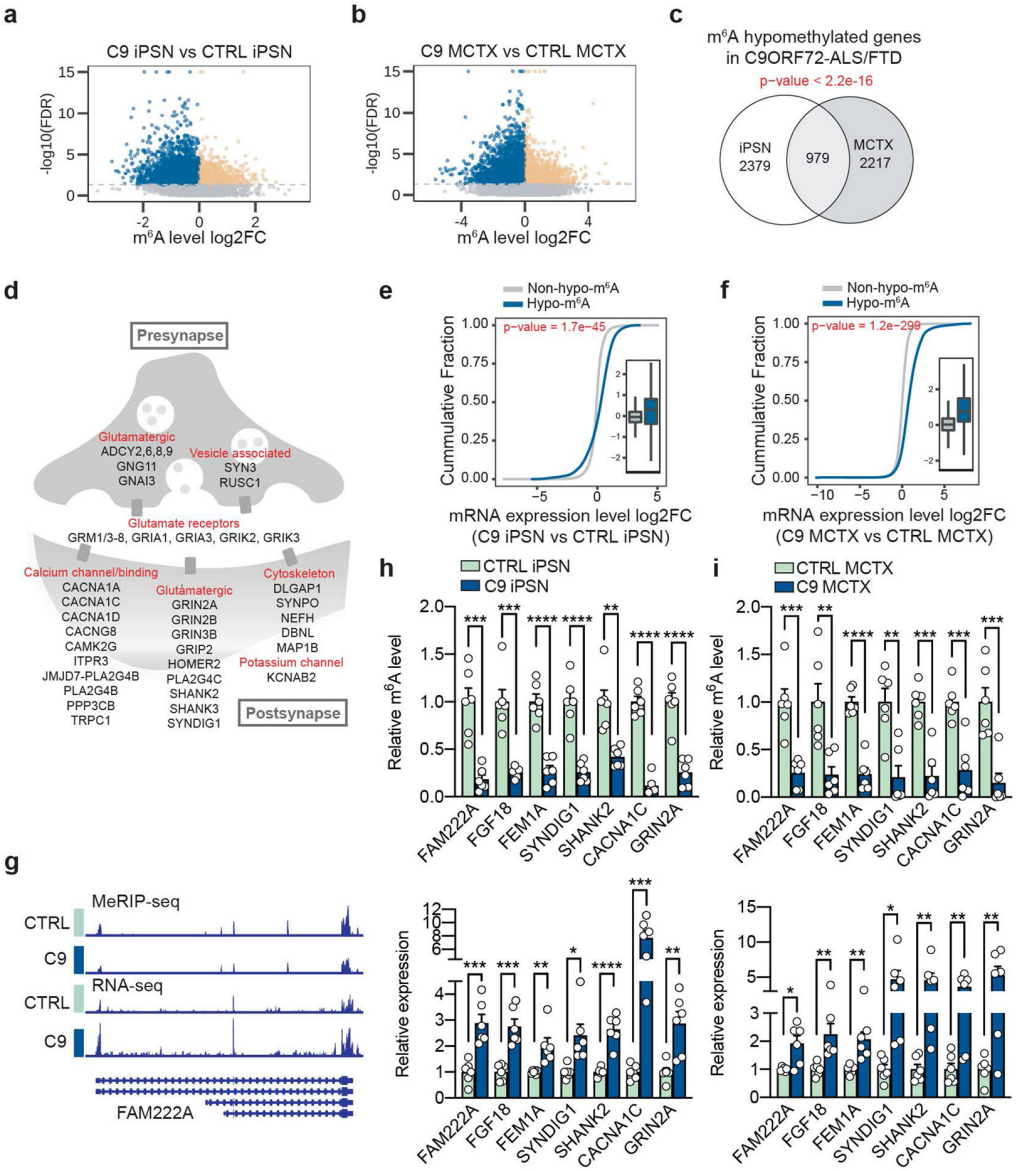
m^6^A hypomethylation dysregulates neuronal gene expression in C9ORF72-ALS/FTD. **a**,**b**, Volcano plot of genes with differential m^6^A level between control and C9ORF72-ALS/FTD in iPSNs (**a**) and in human postmortem motor cortex (**b**). iPSN: *n*=4 individual lines per group, 4037 hypomethylated (blue) *vs* 1494 hypermethylated (orange); Motor cortex (MCTX): *n*=3 individual tissue samples per group, 4146 hypomethylated *vs* 2091 hypermethylated. FDR < 0.01. **c,** Venn diagram depicting the overlaps of hypomethylated genes between iPSN and motor cortex. p-value was calculated by two-tailed Fisher’s exact test. **d,** Schematic of hypomethylated genes related to synaptic functions shared between iPSN and MCTX. **e**,**f**, Cumulative distribution and boxplot (inside) of mRNA expression changes with or without hypo-m^6^A in patient iPSNs (**e**) and motor cortex (**f**). iPSN: *n*=4 individual lines per group; MCTX: *n*=3 individual tissue samples per group. p-values were calculated by a two-tailed non-parametric Wilcoxon-Matt-Whitney test. Box plots indicate the interquartile range with the central line representing the median, and the vertical lines extend to the extreme values in the group. **g**, IGV profiles showing m^6^A signal (generated by comparing MeRIP against input) and RNA-seq reads in the selected mRNA target, *FAM222A.* **h**,**i**, The m^6^A level measured by MeRIP-RT-qPCR (Top) and the relative RNA expression quantified by RT-qPCR (Bottom) of selected transcripts in iPSNs (**h**) and motor cortex (**i**). Points represent individual control or patient iPSN lines (*n*=6 per group) or tissue samples (*n*=6 per group). The isogenic control was set to 1. Data are mean ± s.e.m. *p<0.05, **p<0.01, ***p<0.001, ****p<0.0001, by paired two-tailed *t* test.

Given the prevalence of m^6^A modification in mRNAs, we examined whether the m^6^A reduction leads to global mRNA metabolism changes, which could contribute to neurodegeneration in C9ORF72-ALS/FTD. We performed m^6^A RNA immunoprecipitation (MeRIP) coupled with high-throughput sequencing to map the transcriptome-wide m^6^A sites and compared the differences between control and patients using both iPSNs and postmortem motor cortex (MCTX) samples. About 30,000 m^6^A peaks were identified per group (Extended Data Fig. 5a), with enrichment of the GGACU consensus motif (Extended Data Fig. 5b). As expected, the peaks are abundant in coding sequences (CDS) and 3’ untranslated regions (3’ UTRs), especially enriched near the stop codons (Extended Data Fig. 5c). The C9ORF72-ALS/FTD neurons presented different m^6^A profiles from controls (Extended Data Fig. 5d), and there were significantly more hypomethylated peaks in C9 patient iPSNs and motor cortex tissues (Fig. 2a,b). The hypomethylated genes identified in iPSNs and postmortem motor cortex shared considerable overlaps (Fig. 2c), which were highly enriched in synaptic and neuronal functions (Fig. 2d and Extended Data Fig. 5e). Together, the MeRIP-seq confirmed the global m^6^A reduction and revealed the transcriptome-wide hypomethylation in C9ORF72-ALS/FTD patient iPSNs and postmortem tissues.

### Global m^6^A hypomethylation dysregulates gene expression

**Extended Data Fig. 6:**
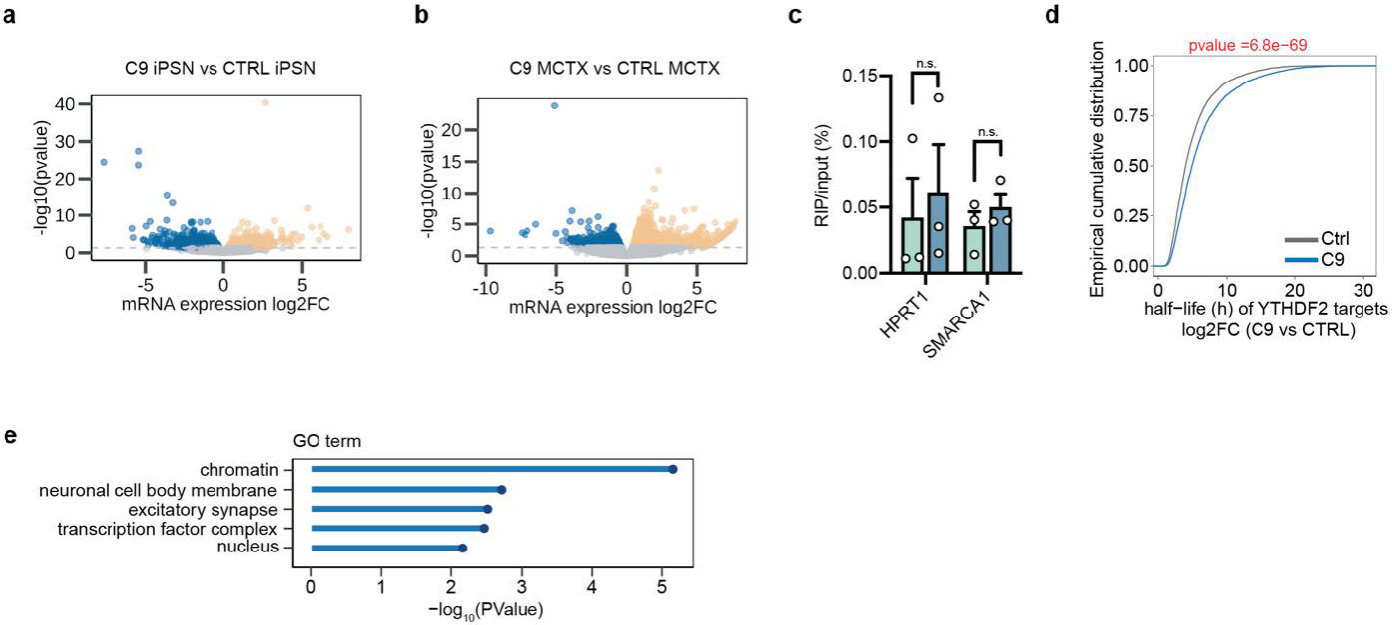
The impact of m^6^A hypomethylation on mRNAs. **a**,**b**, Volcano plot of differentially expressed genes in C9ORF72-ALS/FTD compared to the control, in iPSNs (**a**) and postmortem motor cortex (**b**). Genes with significant changes are represented by orange (upregulated in C9) and blue (downregulated in C9) dots. iPSNs: n=4 individual lines per group, 1212 upregulated vs 1168 downregulated. Motor cortex: n=3 individual tissue samples per group, 1892 upregulated versus 1239 downregulated. p<0.05, two-sided. **c**, YTHDF2-RIP qPCR of selected YTHDF2 mRNA targets identified from the non-YTHDF2 bound RNAs. *HPRT1* and *SMARCA1* mRNAs have no m^6^A modification according to the MeRIP-seq results. Points represent individual iPSN lines. n=3 in each group. Data are mean ± s.e.m. *p*=0.7047 and *p*=0.4001, respectively, by two-tailed *t* test. **d**, Cumulative distribution demonstrating the half-life of YTHDF2 targets between control and C9ORF72-ALS/FTD iPSNs. p-value was calculated by a two-tailed non-parametric Wilcoxon-Matt-Whitney test. **e**, Gene Ontology (GO) enrichment analysis of hypomethylated YTHDF2 binding targets which also showed significantly increased half-life.

**Fig. 3:**
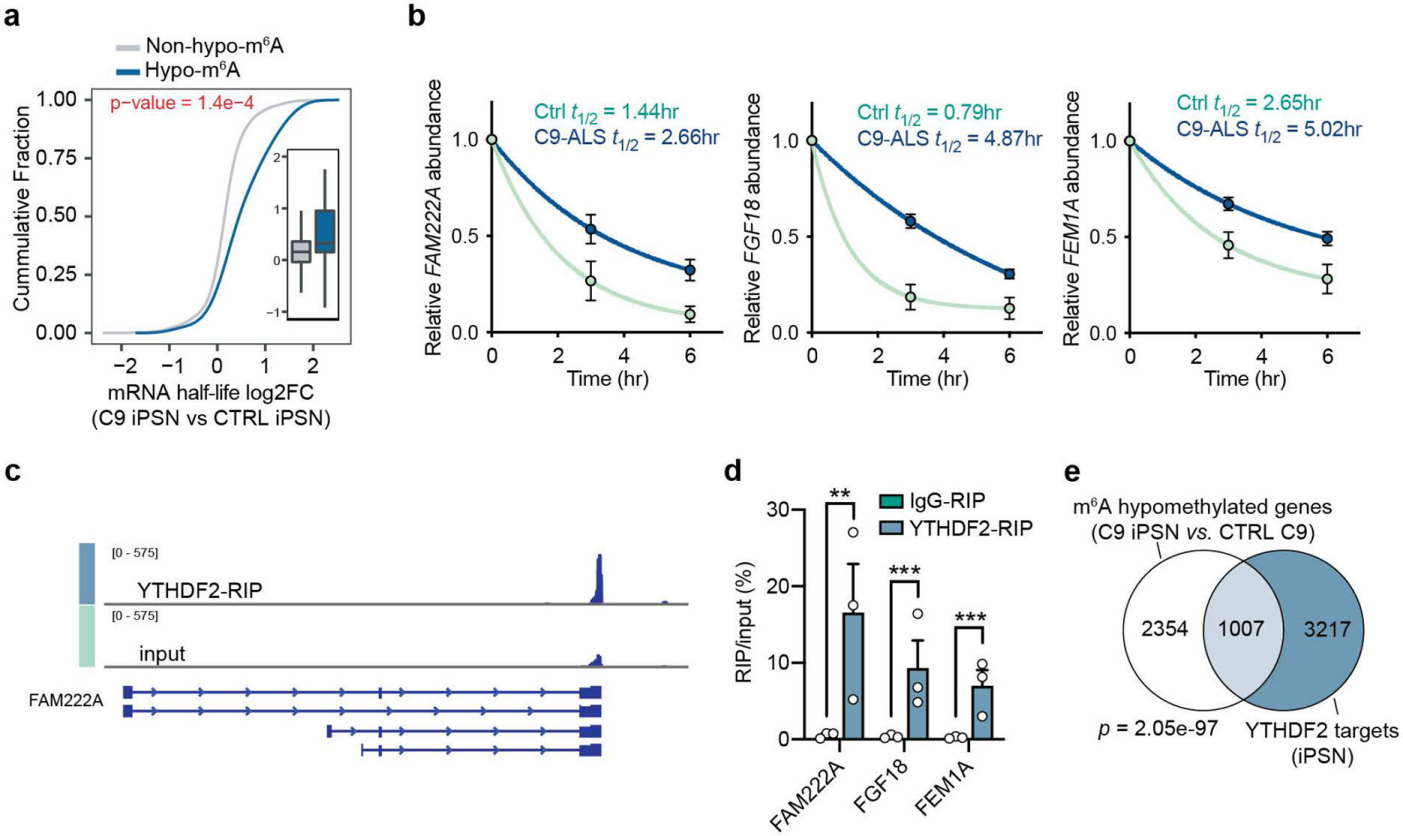
m^6^A hypomethylation increases mRNA half-life via YTHDF2 in iPSNs. **a**, Cumulative distribution and boxplot (inside) of mRNA half-life changes in control and C9ORF72-ALS/FTD iPSNs. n=3 individual lines per group. p-value was calculated by a two-tailed non-parametric Wilcoxon-Matt-Whitney test. Box plots indicate the median, first and third quartiles and range. **b**, Half-life of the representative mRNA targets in control and C9ORF72-ALS/FTD iPSNs. The RNA level was measured by RT-qPCR at each time point and normalized to the data at time 0. *n*=3 individual control or C9ORF72-ALS/FTD iPSN lines. Data are mean ± s.e.m. **c**, IGV profiles showing YTHDF2 binding signal in the selected mRNA target, *FAM222A*. **d**, YTHDF2-RIP qPCR of selected YTHDF2 mRNA targets identified from the YTHDF2-RIP-seq. n=5 control iPSN lines. Points represent individual cell lines. Data are mean ± s.e.m. FAM222A, **p=0.003; FGF18, ***p=0.0001; FEM1A, ***p=0.0006; by two-tailed *t* test. **e**, Venn diagram depicting the overlaps of hypomethylated genes and YTHDF2 targets. p-value was calculated by two-tailed Fisher’s exact test.

We next assessed the potential impact of m^6^A hypomethylation on mRNA expression in C9ORF72-ALS/FTD by RNA-seq analysis (Extended Data Fig. 6a,b). Based on the m^6^A changes in C9 patients (Fig. 2a,b), we categorized transcripts into hypomethylated (hypo-m^6^A) and non-hypomethylated (non-hypo-m^6^A) groups. We found that the transcripts with hypomethylation exhibited significantly upregulated expression levels in C9ORF72-ALS/FTD patient iPSNs and motor cortices, while the non-hypomethylated transcripts tend to show no significant differences between patient and control (Fig. 2e,f). We validated several candidate genes. Indeed, the reduced m^6^A mark on the transcripts correlated with increased gene expression in patients iPSNs and motor cortices (Fig. 2g-i). To examine whether the m^6^A hypomethylation leads to prolonged mRNA half-life that contributes to the gene upregulation, we measured the RNA decay rate in control and patient iPSNs. The neurons were treated with actinomycin D to halt de novo transcription, and RNAs were collected 0, 3, and 6 hours after treatment for RNA-seq to quantify the transcriptome-wide mRNA half-life. We identified that the hypomethylated transcripts in C9ORF72-ALS iPSNs exhibited significantly increased half-life compared to control iPSNs (Fig. 3a,b).

YTHDF2 is an m^6^A “reader” known to facilitate degradation of m^6^A-marked mRNAs in the cytoplasm^13,15^. We performed YTHDF2 RNA immunoprecipitation (RIP) sequencing to identify its binding targets in iPSNs (Fig. 3c,d and Extended Data Fig. 6c). We identified that a significant proportion of the hypomethylated transcripts were bound by YTHDF2 (Fig. 3e), and the half-life of YTHDF2 mRNA targets were significantly increased in the C9ORF72-ALS/FTD iPSNs compared to the control (Extended Data Fig. 6d). Many hypomethylated YTHDF2 target gene transcripts with increased half-life were linked to synapse and neuronal functions (Extended Data Fig. 6e). Altogether, these results reveal that the m^6^A hypomethylation in mRNAs hinders the proper decay of transcripts majorly important for synaptic functions in C9ORF72-ALS/FTD, potentially contributing to neuronal dysfunction and degeneration.

### m^6^A in the repeat-containing intron modulates DPR production

**Fig. 4:**
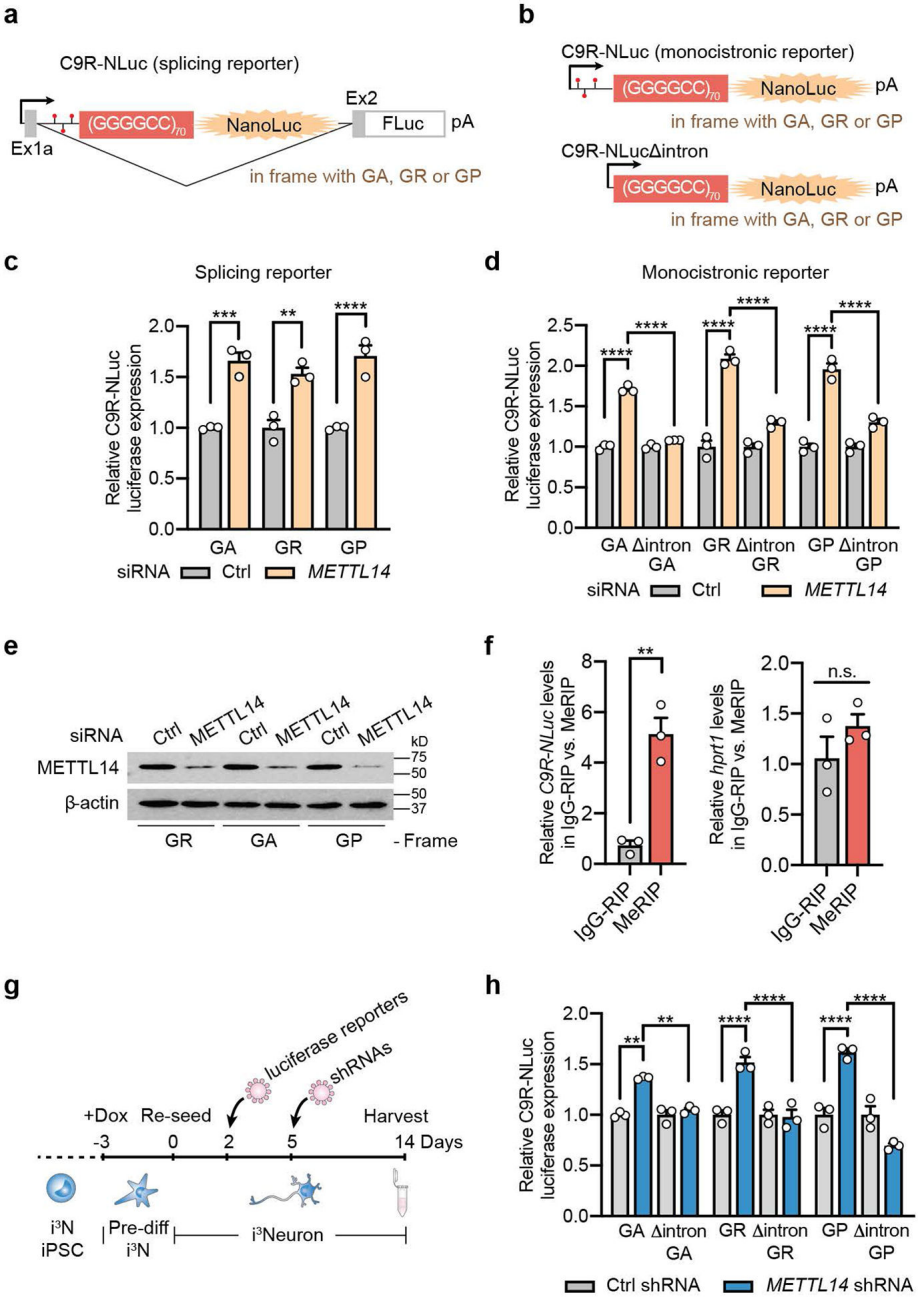
The m^6^A modification in the repeat-containing intron modulates DPR protein production. **a**, Schematic of the bicistronic splicing reporter for (GGGGCC)_70_ RAN translation, which mimics the splicing of *C9* repeat RNA from the endogenous context. Predicted m^6^A sites were indicated as red lollipops in the intron sequence upstream of the repeats. **b**, Schematic of the monocistronic mRNA luciferase reporters for (GGGGCC)_70_ RAN translation with or without the intronic sequence. Predicted m^6^A sites were indicated as red lollipops. **c**, Relative DPR levels were measured by luciferase assay upon METTL14 knockdown in the HeLa Flp-In cells expressing the reporters in (**a**). NLuc signal was normalized to the total protein level in each sample and the relative expression was compared to non-targeting siRNA control. Data are mean ± s.e.m. GA, ***p=0.0002; GR, ** *p*=0.0013; GP, ****p<0.0001, by one-way analysis of variance (ANOVA) with Tukey’s multiple comparisons. **d**, Relative DPR levels were measured by luciferase assay upon METTL14 knockdown in the HeLa Flp-In cells expressing the reporters in (**b**). NLuc signal was normalized to the total protein level in each sample and the relative expression was compared to non-targeting siRNA control. Data are mean ± s.e.m. ****p<0.0001, by one-way analysis of variance (ANOVA) with Tukey’s multiple comparisons. **e**, Western blotting of METTL14 in non-targeting or METTL14 siRNA transfected HeLa Flp-In cells. β-actin was blotted as internal control. **f**, RNA immunoprecipitation by IgG or m^6^A antibody (MeRIP) followed by RT-qPCR of *HPRT1* RNA (left) or *C9* repeat RNA (right) in the HeLa Flp-In C9R-NLuc reporter cells. *HPRT1* RNA does not have m^6^A modification and serves as a negative control. Data are mean ± s.e.m. Points represent three biological replicates. *C9R-NLuc*, **p=0.0028; *HPRT1, p*=0.2584, by two-tailed *t* test. **g**, Timeline for the luciferase reporter experiments in human i^3^Neurons. **h**, Relative DPR levels were measured by luciferase assay upon METTL14 knockdown in the i^3^Neurons expressing the reporters as described in (**g**). n=3 biological replicates. Data are mean ± s.e.m. **p=0.001 and 0.0053, respectively, ****p<0.0001, by one-way analysis of variance (ANOVA) with Tukey’s multiple comparisons. Points represent three biological replicates in each group.

Besides global mRNAs, we also specifically examined the effect of m^6^A reduction on C9ORF72 repeat RNA metabolism. We previously found that the spliced intron containing the expanded (GGGGCC)n repeats is exported to cytoplasm and subjected to the repeat-associated non-AUG (RAN) translation in all the three reading frames (poly-GA, poly-GP, poly-GR)^22,23^. We identified m^6^A methyltransferase components as suppressors of DPR production by an unbiased CRISPR screen^5^. We first validated the effect using the established HeLa Flp-In dual luciferase reporter systems, in which the NanoLuc luciferase is generated through RAN translation of C9ORF72 GGGGCC repeats (C9R-NLuc)^5,22^. In both the splicing reporter, in which the C9R-NLuc is located in the *C9ORF72* intron (Fig. 4a), and the monocistronic mRNA reporter (Fig. 4b), knockdown of METTL14 significantly increased the DPR proteins from all the reading frames (Fig. 4c-e). This suggests the m^6^A modification can modulate DPR production, and this effect is independent of splicing.

**Extended Data Fig. 7:**
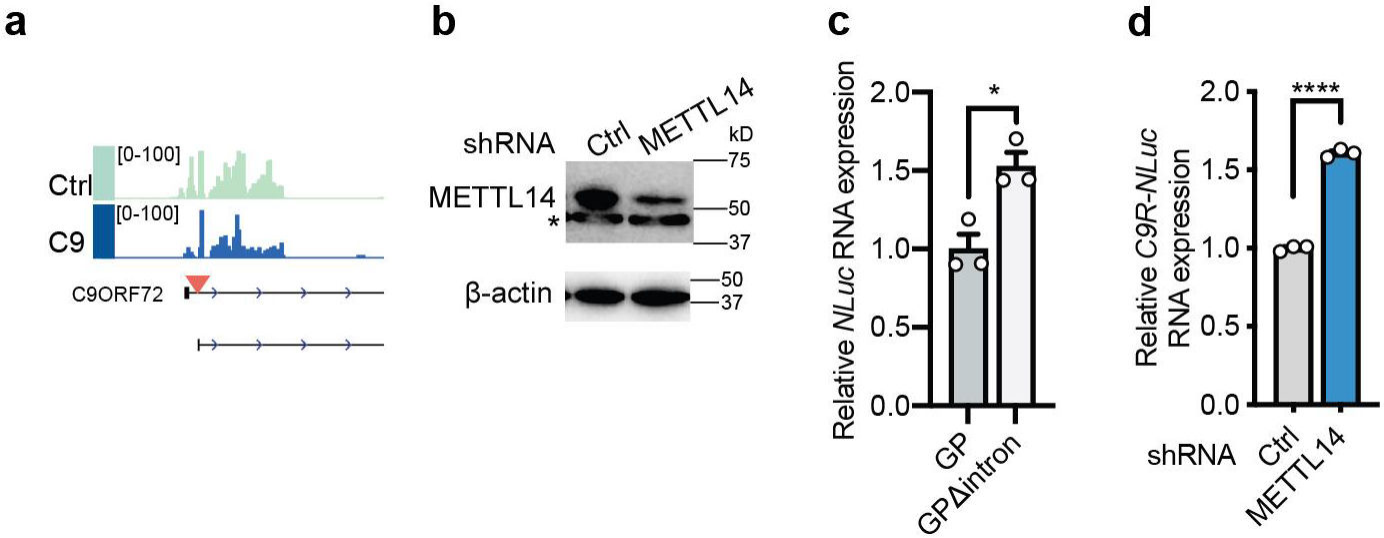
m^6^A regulates *C9ORF72* repeat-containing RNA. **a**, IGV profile showing the m^6^A peaks (generated by comparing MeRIP against input) in control and C9ORF72-ALS/FTD iPSNs. Red arrow head represents the repeat expansion. **b**, Western blotting of METTL14 in control or METTL14 knockdown i^3^Neurons. *non-specific band. **c**, The relative basal expression of *NLuc* RNA in the HeLa Flp-In cells expressing the reporters as described in Fig. 4b. *p=0.015 by two-tailed *t* test. **d**, The relative expression level of *C9R-NLuc* RNA upon knockdown of METTL14 in i^3^Neurons, measured by RT-qPCR and normalized to non-targeting shRNA control. ****p<0.0001 by two-tailed *t* test. (**c,d**) Points represent three biological replicates. Data are mean ± s.e.m.

The intronic sequence upstream of the repeat expansion contains several m^6^A consensus motifs. Indeed, the MeRIP-seq revealed m^6^A peaks in the *C9ORF72* intron regions surrounding the repeats (Extended Data Fig. 7a). MeRIP coupled with RT-qPCR showed enrichment of the *C9R-NLuc* RNA but not the negative control (Fig. 4f). Furthermore, deletion of the intronic sequence (Δintron) upstream of the repeats eliminated the DPR upregulation induced by METTL14 reduction (Fig. 4b,d). Altogether, this reveals that the m^6^A modification in the C9 intron can regulate the DPR production from the repeat-containing RNA.

We further validated the results in human i^3^Neurons. We expressed the luciferase reporters and shRNA targeting METTL14 in differentiated i^3^Neurons via lentiviral transduction (Fig. 4g). Similar to the observation in HeLa cells, reduction of METTL14 increased the levels of all the three DPR proteins, and this effect was diminished in the Δintron mutants (Fig. 4h, Extended Data Fig. 7b). This demonstrates that the m^6^A modification-mediated DPR regulation mechanism is conserved in neurons.

### YTHDC1-NEXT regulates the stability of *C9ORF72* repeat RNA

**Extended Data Fig. 8:**
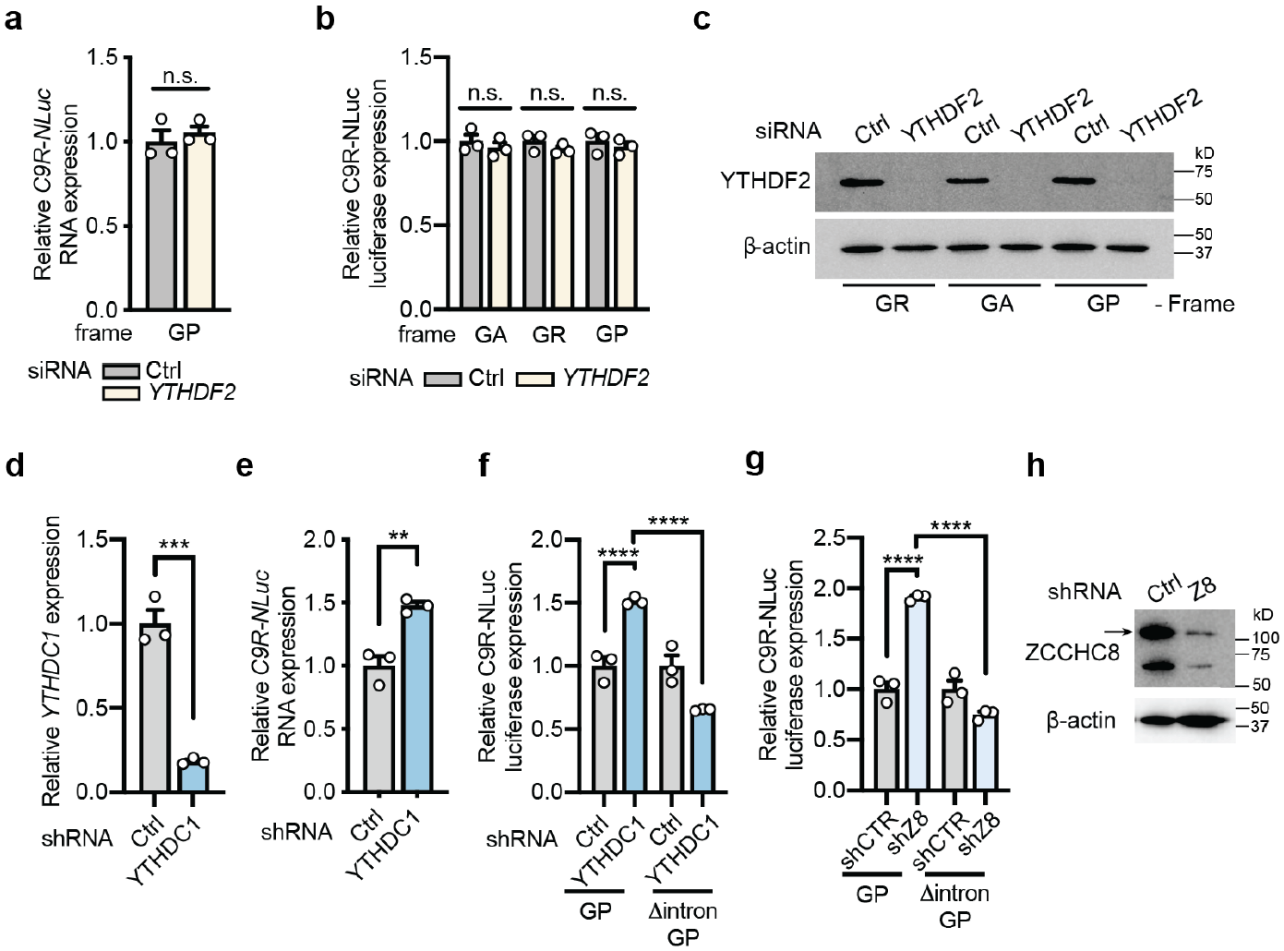
The turnover of *C9ORF72* repeat-containing RNA is regulated by m^6^A in i^3^Neurons. **a**, RT-qPCR of C9 repeat RNA expression from the C9R-NLuc HeLa reporter upon knockdown of YTHDF2. *p*=0.5418 by two-tailed *t* test. **b**, Relative DPR level was measured by luciferase assay upon YTHDF2 knockdown in the C9R-NLuc HeLa reporter. **c**, Western blotting of YTHDF2 in control or YTHDF2 knockdown HeLa Flp-In cells. *p*=0.9456, 0.8801 and 0.9731, respectively, by one-way analysis of variance (ANOVA) with Tukey’s multiple comparisons. **d,e**, The relative expression level of *YTHDC1* RNA (**d**) or *C9R-NLuc* RNA (**e**) upon knockdown of YTHDC1 by shRNA in i^3^Neurons. **p=0.0046, ***p=0.0006 by two-tailed *t* test. **f,g**, Relative DPR levels were measured by luciferase assay upon YTHDC1 knockdown (f) or ZCCHC8 knockdown (g) in the i^3^Neurons expressing the reporters in Fig. 4b. NLuc signal was normalized to the total protein level in each sample and the relative expression was compared to non-targeting shRNA control. ****p<0.0001 by one-way analysis of variance (ANOVA) with Tukey’s multiple comparisons. **h**, Western blotting of ZCCHC8 in control or ZCCHC8 knockdown i^3^Neurons. Arrow points the expected size of ZCCHC8. (**a,b,d-g**) Data are mean ± s.e.m. Points represent three biological replicates.

**Extended Data Fig. 9:**
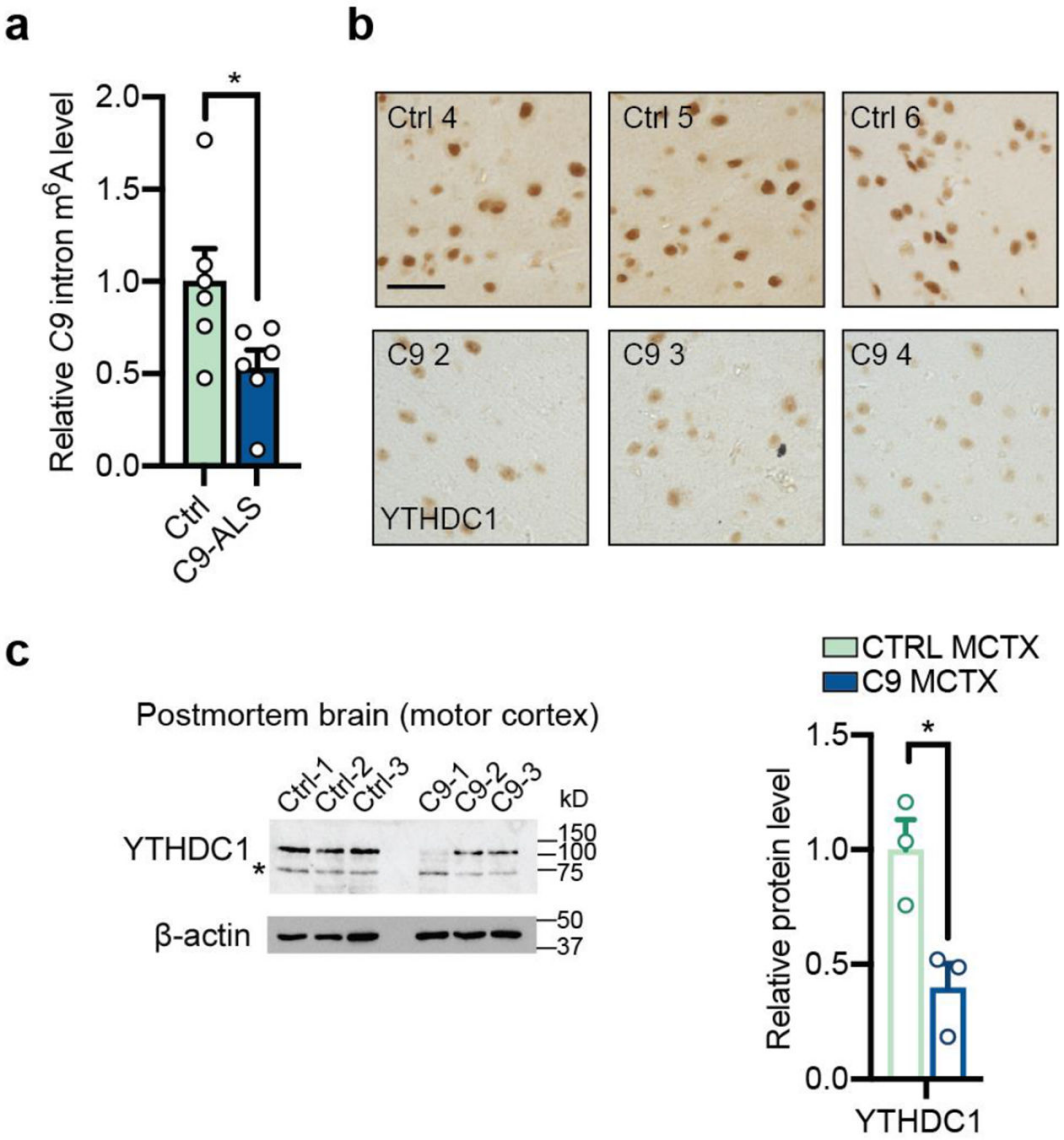
m^6^A hypomethylation of the endogenous C9 repeat-containing intron and YTHDC1 expression in the postmortem brain tissues. **a**, MeRIP coupled with RT-qPCR of endogenous C9 repeat-containing intronic RNA in control and C9ORF72-ALS/FTD iPSNs. Points represent individual cell lines. n=6 per group. *p=0.0427. **b**, Representative IHC staining of YTHDC1 at temporal cortex section of control and C9ORF72-ALS/FTD samples. Scale bar = 50μm. **c**, Western blotting (left) and quantification (right) of YTHDC1 using postmortem motor cortex tissue samples. n=3 individuals per group. *Non-specific bands. *p=0.0246. (**a,c**) Data are mean ± s.e.m. Points represent individual control or patient samples. p-values were calculated by two-tailed *t* test.

**Fig. 5:**
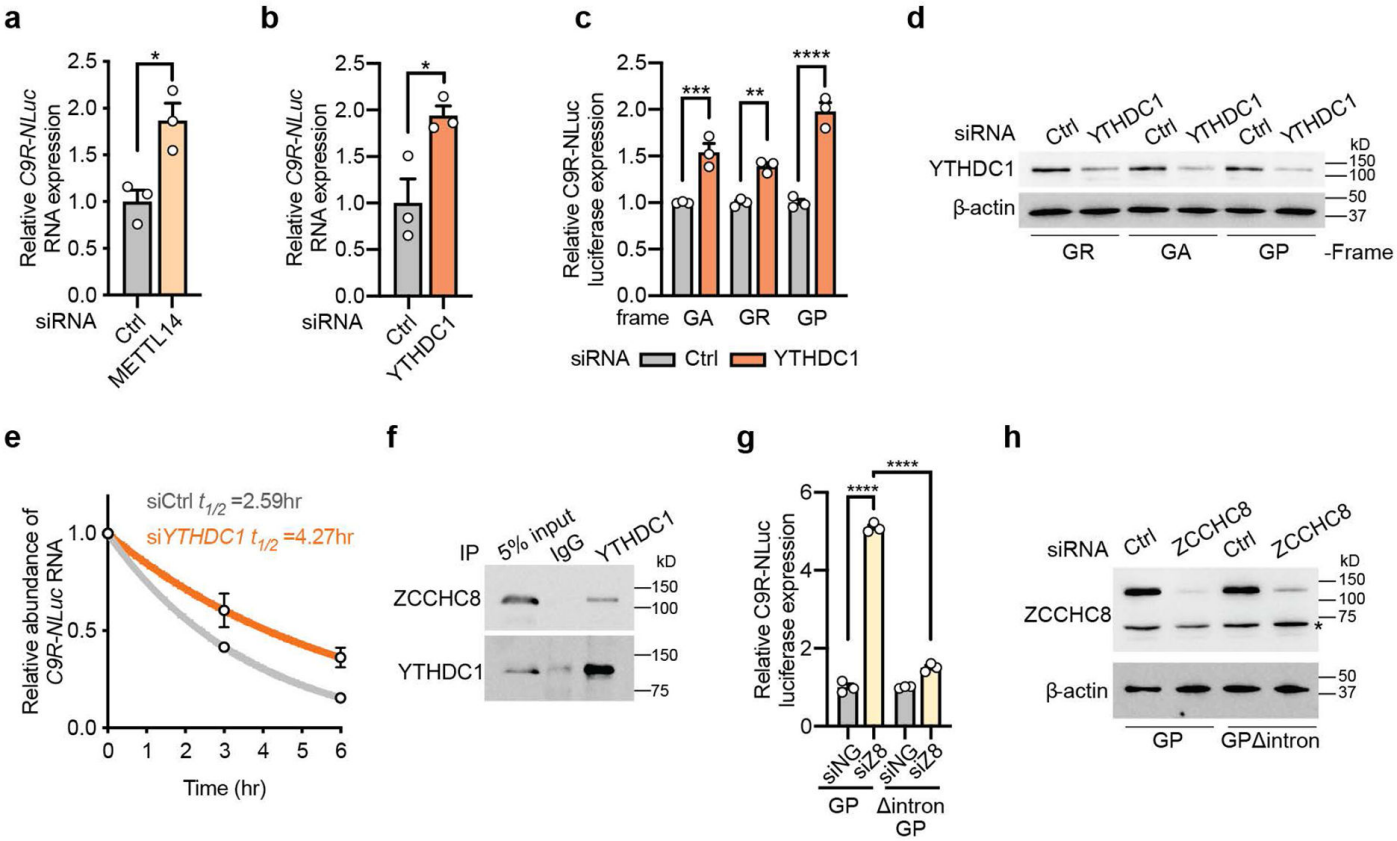
YTHDC1 regulates the stability of *C9ORF72* repeat-containing intron RNA via the NEXT decay pathway. **a**,**b**, The relative expression level of *C9R-NLuc* RNA upon knockdown of METTL14 (**a**) or YTHDC1 (**b**) measured by RT-qPCR and normalized to non-targeting siRNA control. *p=0.0175 (a), *p=0.0283 (b) by two-tailed *t* test. **c**, Relative DPR levels were measured by luciferase assay upon YTHDC1 knockdown. NLuc signals were normalized to the total protein level. **p=0.0062, ***p=0.0004, ****p<0.0001 by one-way analysis of variance (ANOVA) with Tukey’s multiple comparisons. **d**, Western blotting of YTHDC1 in non-targeting or YTHDC1 siRNA transfected HeLa Flp-In cells. **e**, Half-life of *C9* repeat RNA in HeLa C9R-NLuc reporter cells transfected with YTHDC1 siRNA or non-targeting control. The RNA level was measured by RT-qPCR at each time point and normalized to the data at time 0. **f**, Immunoprecipitation was performed in human iPSNs with YTHDC1 antibody or IgG control. The input and precipitated proteins were immunoblotted with YTHDC1 and ZCCHC8 antibodies. *n* = 3 biological replicates. **g**, Relative DPR levels were measured by luciferase assay upon ZCCHC8 knockdown. ****p<0.0001 by one-way analysis of variance (ANOVA) with Tukey’s multiple comparisons. **h**, Western blotting of ZCCHC8 in control or ZCCHC8 knockdown HeLa Flp-In cells. *non-specific band. (**a-c**,**e**,**g**) Data are mean ± s.e.m. Points represent three biological replicates at each condition.

To decipher the molecular mechanism of the DPR regulation by m^6^A modification, we examined the repeat-containing RNA level. There was increased *C9R-NLuc* RNA in the Δintron reporter cells compared to the wild type control (Extended Data Fig. 7c), suggesting the m^6^A modification in the intron modulates the repeat RNA accumulation. Indeed, the *C9R-NLuc* RNA was upregulated upon METTL14 knockdown in both HeLa and i^3^Neuron reporter systems (Fig. 5a and Extended Data Fig. 7d), consistent with the DPR changes (Fig. 4d,h). As the steady-state RNA level can be influenced by the RNA turnover rate, we examined two m^6^A “readers” with well-documented RNA decay function: YTHDC1, primarily responsible for the degradation of non-coding RNAs in the nucleus^24^; and YTHDF2, predominantly in the cytoplasm for mRNA decay^13,15^. We found that knockdown of YTHDF2 did not induce any changes (Extended Data Fig. 8a-c), but knockdown of YTHDC1 significantly increased the RNA and DPR levels of C9R-NLuc in both HeLa cells and i^3^Neurons (Fig. 5b-d and Extended Data Fig. 8d-f), consistent with the effect by METTL14 reduction (Fig. 4d,h,5a and Extended Data Fig. 7d). The elevation of DPRs by YTHDC1 knockdown was abolished in the intron-deletion mutant reporter (Extended Data Fig. 8f). In addition, the upregulated RNA level was in line with the increased half-life (Fig. 5e). Co-immunoprecipitation showed that YTHDC1 interacts with ZCCHC8, the core component of the Nuclear Exosome Targeting (NEXT) complex^25^ in iPSNs (Fig. 5f). Knockdown of ZCCHC8 significantly increased DPR production, and the effect was dependent on the intronic sequence preceding the repeats (Fig. 5g,h and Extended Data Fig. 8g,h). Altogether, the results from both HeLa cells and i^3^Neurons support that YTHDC1 mediates the decay of the m^6^A-marked C9 intron RNA by targeting to the NEXT complex. In C9ORF72-ALS/FTD, the hypomethylation of the repeat-containing intronic RNA (Extended Data Fig. 7a,9a) and the reduction of YTHDC1 protein (Extended Data Fig. 9b,c) could synergistically decelerate the RNA degradation, contributing to the increased accumulation of repeat RNA and DPRs with various toxicity.

### m^6^A restoration rescues disease-related phenotypes

**Extended Data Fig. 10:**
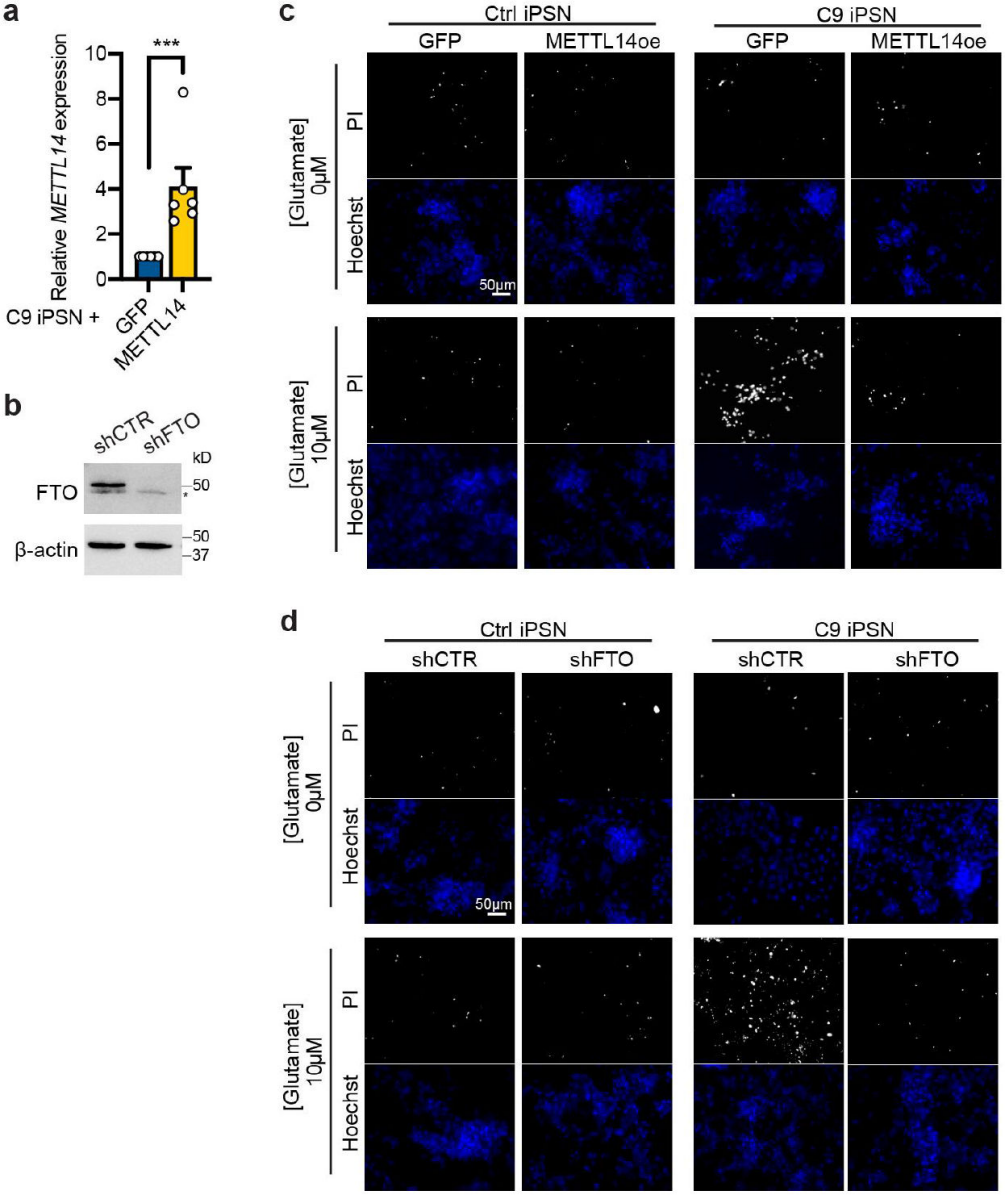
m^6^A restoration reduces glutamate-induced excitotoxicity in C9ORF72-ALS/FTD iPSNs. **a**, RT-qPCR of *METTL14* in C9ORF72-ALS/FTD iPSNs expressing exogenous METTL14 or GFP control. Data are mean ± s.e.m. Points represent individual cell lines. n=6 in each group. ***p=0.0006 by paired two-tailed *t* test. **b**, Western blotting of FTO in C9ORF72-ALS/FTD iPSNs upon knockdown of FTO by shRNA. *non-specific band. **c**,**d**, Representative images of Hoechst and propidium iodide (PI) staining of iPSNs in the glutamate-induced excitotoxicity assay. C9ORF72-ALS/FTD iPSNs were infected with lentivirus overexpressing METTL14 or GFP as negative control (c), or expressing FTO shRNA or non-targeting control shRNA (d).

**Fig. 6:**
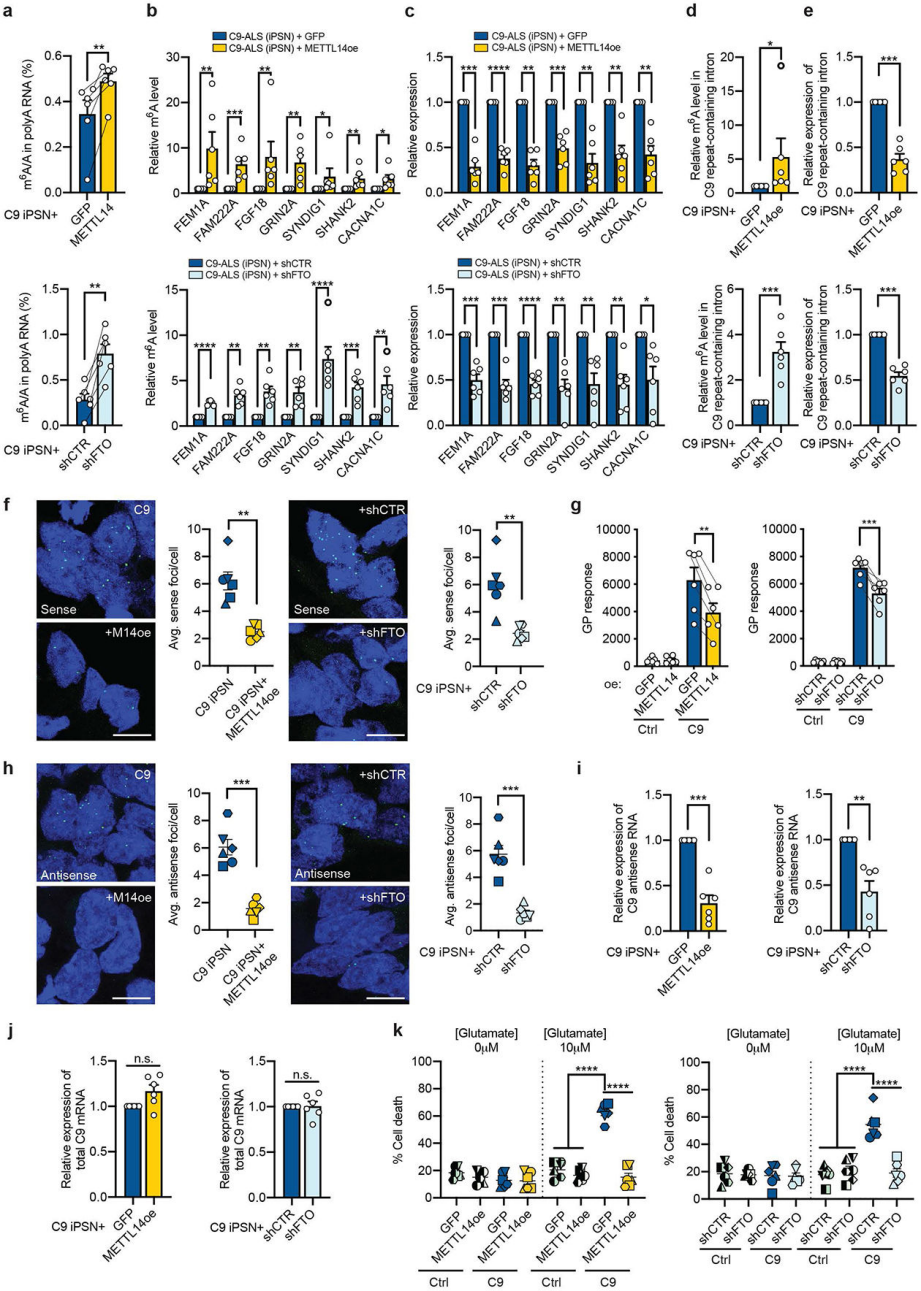
m^6^A restoration rescues disease-related phenotypes in C9ORF72-ALS/FTD patient iPSNs. C9ORF72-ALS/FTD iPSNs were infected with lentivirus overexpressing METTL14 or GFP as negative control, or with lentivirus expressing FTO shRNA or non-targeting control shRNA. Data are from six patient iPSN lines. Points represent individual lines. **a**, UHPLC-QQQ-MS/MS quantification of the m^6^A/A ratio in poly-A RNAs from iPSNs with METTL14 overexpression (Top) or FTO knockdown (Bottom). **p=0.0043 and 0.0012, respectively, by paired two-tailed *t* test. **b**, The m^6^A level of representative mRNA targets was examined by MeRIP coupled with RT-qPCR. (Top) Overexpression of METTL14. (Bottom) Knockdown of FTO. *p<0.05, **p<0.01, ***p<0.001, ****p<0.0001, by paired two-tailed *t* test. **c**, The RNA expression of the targets was examined by RT-qPCR. (Top) Overexpression of METTL14. (Bottom) Knockdown of FTO. *p<0.05, **p<0.01, ***p<0.001, ****p<0.0001, by paired two-tailed *t* test. **d**, Relative m^6^A level in the *C9* repeat-containing intron was measured by MeRIP-RT-qPCR. *p=0.0319, ***p=0.0005, by paired two-tailed *t* test. **e**, Relative RNA expression of *C9* repeat-containing intron was measured by RT-qPCR. ***p=0.0001 by paired two-tailed *t* test. **f**,**h**, Representative images and quantification of FISH experiment to examine the sense (**f**) and antisense (**h**) RNA foci changes in C9ORF72-ALS/FTD iPSNs upon METTL14 overexpression (Left) or FTO knockdown (Right). Scale bar, 10μm. **p=0.0044 and 0.0058, respectively (f); ***p=0.0001 and 0.0009, respectively (h), by paired two-tailed *t* test. **g**, Poly-GP protein level was measured by ELISA. (Left) Overexpression of METTL14. (Right) Knockdown of FTO. **p=0.0024, ***p=0.001, by paired two-tailed *t* test. **i**, RT-qPCR of endogenous antisense repeat RNA expression in C9ORF72-ALS/FTD iPSNs upon METTL14 overexpression (Left) or FTO knockdown (Right). **p=0.0046, ***p=0.0005, by paired two-tailed *t* test. **j**, RT-qPCR of total C9 mRNA expression in C9ORF72-ALS/FTD iPSNs upon METTL14 overexpression (Left) or FTO knockdown (Right). *p*=0.0617 and 0.9238, respectively. **k**, Glutamate-induced excitotoxicity in control and C9ORF72-ALS/FTD iPSNs overexpressing GFP *vs* METTL14, or expressing control shRNA *vs* FTO shRNA. Different shapes represent different iPSN lines from 6 control and 6 patients. ****p<0.0001 by paired two-tailed *t* test. (**a-k**) Data are mean ± s.e.m. Points represent individual cell lines.

We next examined whether elevating the m^6^A level could rescue the disease-related phenotypes in C9ORF72-ALS/FTD patient iPSNs. We either overexpressed the METTL14 methyltransferase (Extended Data Fig. 10a) or knocked down the FTO demethylase (Extended Data Fig. 10b) via lentiviral transduction in neurons differentiated from patient iPSCs. Both approaches significantly increased the m^6^A level (Fig. 6a). Elevation of m^6^A on the hypomethylated mRNA transcripts reduced their abnormal upregulation in C9ORF72 iPSNs (Fig. 6b,c), indicating the restoration of the RNA homeostasis in patient neurons. Furthermore, the m^6^A upregulation on the C9ORF72 repeat-containing intron RNA (Fig. 6d) also effectively decreased the repeat RNA abundance, measured by both RT-qPCR of the intron region (Fig. 6e) and RNA fluorescence in situ hybridization (FISH) targeting the sense GGGGCC repeats (Fig. 6f). Accordingly, the endogenous poly-GP level was also reduced in patient iPSNs (Fig. 6g).

Additionally, we examined the antisense repeat-containing RNA, which is produced by bi-directional transcription at the repeat expansion region and could potentially contribute to the pathogenesis^26-30^. Using both RNA FISH and stranded RT-qPCR^27,31^, we found that the antisense RNA, similar to the sense repeat-containing intron, was also reduced by METTL14 overexpression or FTO knockdown in patient iPSNs (Fig. 6h,i). Notably, the total *C9ORF72* mRNA level was not affected by m^6^A changes (Fig. 6j). This reveals the exciting opportunity to diminish both strands of the potentially toxic repeat RNAs and derived DPRs without influencing the C9ORF72 protein expression by manipulating one candidate genetic modifier.

Finally, we tested whether increasing m^6^A can improve neuron survival. C9ORF72-ALS/FTD iPSNs are more susceptible to glutamate-induced excitotoxicity compared to controls^32^. We quantified the percentage of neuron death after glutamate treatment and found that either METTL14 overexpression or FTO knockdown was sufficient to alleviate the glutamate-induced cell death of C9ORF72 iPSNs and largely improved neuron survival (Fig. 6k and Extended Data Fig. 10c,d). These results support the m^6^A pathway as a reversible target that could provide beneficial effects to C9ORF72 repeat expansion-linked neurodegeneration.

### FTO inhibitor treatment mitigates disease-linked phenotypes

**Fig. 7:**
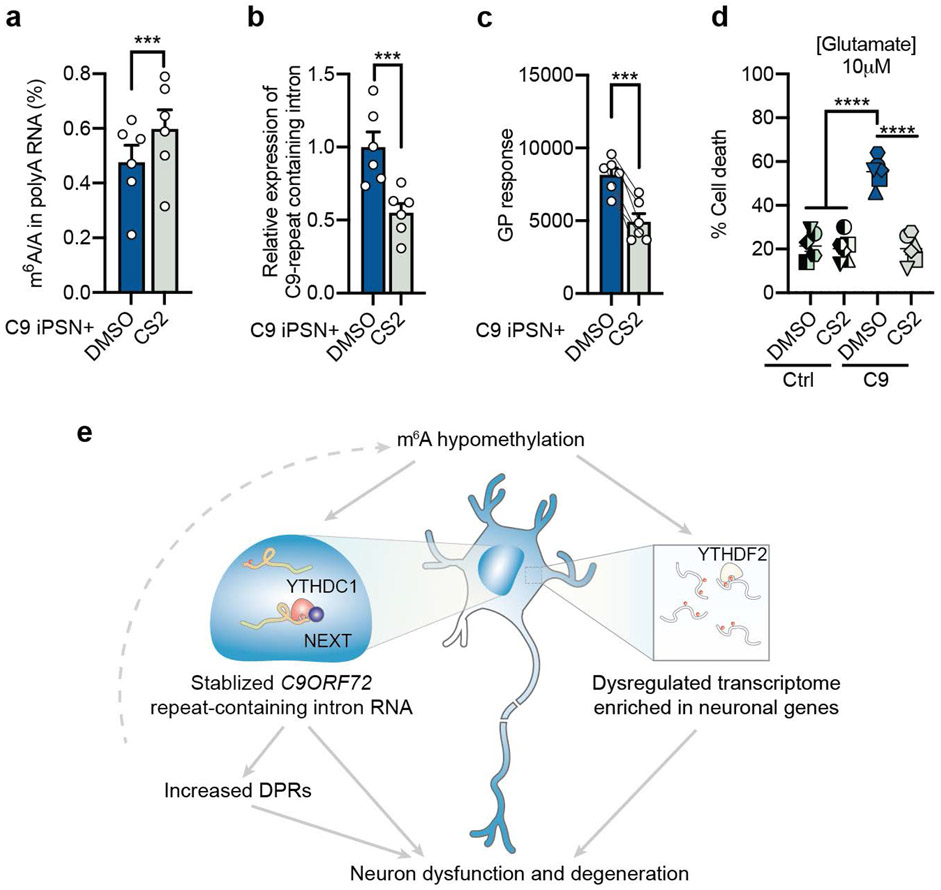
FTO inhibitor treatment mitigates disease phenotypes. **a**, UHPLC-QQQ-MS/MS quantification of the m^6^A/A ratio in poly-A RNAs from iPSNs with CS2 (500nM) treatment for 7 days. ***p=0.0003 by paired two-tailed *t* test. **b,c**, Relative expression of *C9* repeat-containing intron RNA (**b**) and poly-GP protein level (**c**) in C9ORF72-ALS/FTD iPSNs after treatment with DMSO control or FTO inhibitor, CS2 (500nM), for 7 days. ***p=0.0009 (b) and ***p=0.0001 (c), by paired two-tailed *t* test. **d**, Glutamate-induced excitotoxicity in control and C9ORF72-ALS/FTD iPSNs after DMSO or CS2 treatment. Different shapes represent different iPSN lines. ****p<0.0001 by paired two-tailed *t* test. **e**, A schematic model showing how global m^6^A hypomethylation in C9ORF72-ALS/FTD impairs neuronal function and results in neuronal death by stabilizing *C9ORF72* repeat RNA and dysregulating neuronal gene expression. Points represent individual patient iPSN lines. *n* = 6 in each group. (**a-d**) Data are mean ± s.e.m.

Small molecule approaches to inhibit the demethylase activity is another way to elevate m^6^A levels. Several small molecule inhibitors of FTO have been developed and reported to exhibit anti-tumor efficacy^33-35^. In particular, CS2 is the latest generation of FTO inhibitor, which showed high potency on FTO inhibition and promising anti-cancer effect^34^. Treatment of C9ORF72-ALS/FTD patient iPSNs with CS2 increased the total m^6^A level on mRNA (Fig. 7a), reduced repeat RNA and DPR levels (Fig. 7b,c), and significantly improved neuron survival (Fig. 7d). This highlights the therapeutic potential of targeting FTO in the neurodegeneration disease.

## Discussion

Here, we found that the expression of the two m^6^A methyltransferases METTL3 and METTL14 is abnormally reduced, triggered by the repeat-containing RNA. On the one hand, the abnormal m^6^A reduction led to the transcriptome-wide mRNA dysregulation with significant enrichment in synaptic activity and neuronal functional pathways. On the other hand, the reduced m^6^A mark on the repeat-containing RNA disturbed the RNA decay, therefore enhancing the accumulation of toxic repeat RNA and DPRs, which further promote m^6^A dysregulation, forming a positive feedback loop. Strategies to elevate the m^6^A RNA modification level have the therapeutic potential in C9ORF72-ALS/FTD, as this can both reduce the toxic DPR/RNA repeats and rescue a broad spectrum of post-transcriptional dysregulation in patient neurons. Thus, this work provides evidence of m^6^A epitranscriptomic dysregulation as a previously unknown pathogenic mechanism of C9ORF72-ALS/FTD (Fig. 7e)

The nervous system has extremely complex RNA processing regulation, including alternative splicing, polyadenylation, transport, stability and translation. Epitranscriptomic regulation recently emerges to play important roles in modulating the functions and structures of RNAs in a spatiotemporal manner and therefore fine-tuning the accuracy of gene expression^36^. Several studies demonstrated the important roles of m^6^A in regulating brain function. Loss of m^6^A methyltransferases in *Drosophila* leads to severe locomotion defects in orientation, walking speed and activity due to impaired neuronal function^37^. Mice with m^6^A pathway loss-of-function displayed multiple brain defects, including smaller brains, weakened spatial learning and memory, and impaired synaptic transmission, long-term potentiation and axon regeneration^17,38^. This suggests the importance of the m^6^A-mediated epitranscriptome in neural function, but there were limited studies on its contribution to human neurological or neurodegenerative diseases.

We found that the global m^6^A reduction leads to transcriptome-wide hypomethylation and dysregulated gene expression in C9ORF72-ALS/FTD patients. The hypomethylated genes in C9ORF72-ALS/FTD are enriched in neuronal functions and synaptic pathways. In particular, many genes are linked to glutamatergic synapses and calcium signaling pathways. This could potentially contribute to the underlying molecular mechanism of hyperexcitability and increased vulnerability to excitotoxicity of patient neurons^32,39,40^. Indeed, METTL14 deletion has been found to result in increased neuronal excitability in striatonigral neurons, through lowered current threshold of action potential generation^41^.

There are several hypotheses of the molecular mechanisms on how C9ORF72 repeat expansion leads to neurodegeneration. The haploinsufficiency of the C9ORF72 protein function might contribute to the disease progression, especially via microglia-mediated deficits^42,43^. But more evidence points to the driving toxicity from the abnormally accumulated sense or/and antisense repeat RNAs, and their encoded DPRs. We now identified that both strands of repeat-containing RNAs can be regulated by the m^6^A-mediated RNA degradation. Elevating the m^6^A level (such as by expressing the methyltransferase METTL14 or by reducing the demethylase FTO) can directly decrease the RNA repeats of both strands, the major upstream toxicity-initiating factors, therefore rescue the overall downstream defects and improve neuronal health. Importantly, the *C9ORF72* mRNA level was not affected by m^6^A, thus the normal protein function will not be affected by this strategy. Furthermore, as the m^6^A RNA modification is decreased in patients, this approach will restore the m^6^A level and rescue the global hypomethylation-mediated dysfunction of essential neuronal genes. All these advantages support the premise of targeting the m^6^A pathway as a novel therapeutic strategy in C9ORF72-ALS/FTD. It will be interesting to test the rescue efficacy *in vivo* using appropriate C9 mouse models in future studies.

It is also noteworthy that the overexpression of the C9 expanded repeats, rather than the DPRs produced by randomized codons, induced METTL3/14 expression decrease. Although the mechanism by which the repeat expansion elicited reduction of METTL3/14 expression remains to be determined, our data emphasize the role of repeat expansion-mediated RNA toxicity in disease-related neuronal deficits. Many recent studies focused on various mechanisms of DPR-mediated neurotoxicity^44^. The repeat RNA-induced toxicity could also play important roles in disease pathogenesis. It is generally believed that the repeat RNA could sequester specific RBPs from their normal targets, therefore perturb their physiological functions. Multiple RBPs have been proposed to be bound and disturbed by the (GGGGCC)n repeat RNA^45,46^, such as ADARB2^32^, hnRNPs^47-49^, and Pur-α^49,50^. However, the pathophysiological contribution of many candidate RBPs remains elusive. Our study reveals a novel mechanism of repeat RNA-mediated toxicity - disturbing the global RNA stability homeostasis via m^6^A modification. Besides the effect on mRNAs, the m^6^A reduction also leads to stabilization of repeat-containing intron RNA, which provides translation substrate for increased production of DPRs. Therefore, this repeat RNA toxicity mechanism might be an early event that can be targeted to reduce both RNA and DPR mediated downstream defects.

In addition, it is noted that the core components of the m^6^A writer complex are also widely reduced in sporadic ALS from analysis of almost one hundred sporadic ALS iPSN lines and seven postmortem tissues (Extended Data Fig. 3). This implicates the possible broader contribution of the m^6^A-mediated RNA dysregulation in sporadic ALS as well. A recent study reported m^6^A hypermethylation in spinal cord of four sporadic ALS patients using a microarray method^51^. Considering the complexity of sporadic disease, screening m^6^A levels in a large number of sporadic ALS cases by using direct UHPLC-QQQ-MS/MS may be necessary. Future development of new techniques to reveal spatial or single-cell m^6^A changes will also help elucidate m^6^A dysregulation in diseases with heterogeneous pathology. Interestingly, the m^6^A pathway was also implicated in other neurodegenerative diseases. METTL3/14 expression and the m^6^A level has been reported to be reduced in the neurons or brains of Alzheimer’s Disease (AD) patients^52,53^, 5×FAD mouse model^54^, and 6-OHDA induced rat model of Parkinson's Disease^55^. A recent study reported that treating primary neurons with Aβ oligomers causes reduced METTL3 expression^52^. Although the molecular mechanisms of the m^6^A dysregulation remain elusive, these studies suggest the epitranscriptome dysregulation as a compelling convergent pathogenic pathway underlying neurodegenerative diseases. Further work is needed to elucidate the mechanisms how the expression of m^6^A writer or eraser proteins is altered, which could provide more therapeutic options to rescue the epitranscriptome deficits in neurodegeneration. Overall, our study highlights the significance of understanding RNA modification-mediated pathogenic mechanism and opens new avenues of developing novel epitranscriptomic therapy in neurodegenerative diseases.

## Methods

### Plasmids

To delete the intron sequences upstream of the GGGGCC repeats, we used HindIII and BssHII to cut out the upstream intron sequences in the monocistronic C9R-NLuc luciferase reporters for (GGGGCC)_70_ RAN translation^5,22^. For the lentiviral C9R-NLuc reporters, the (GGGGCC)_70_-NLuc fragments from the monocistronic mRNA luciferase reporters were cut out by PmeI and XhoI, and subcloned into the lentiviral vector under the EF1a promoter via SbfI (blunted by NEB Quick Blunting Kit) and XhoI. For lentiviral METTL14 overexpression, the Flag-METTL14 fragment was cut out by EcoRI and BamHI from the pcDNA3-Flag-METTL14 (Addgene# 53740), and subcloned into the lentiviral vector under the EF1a promoter via the same restrictive enzyme sites. For the design and cloning of lentiviral METTL14 and ZCCHC8 shRNAs, we followed the Addgene pLKO.1 shRNA cloning protocol (https://www.addgene.org/protocols/plko/). In brief, oligos containing the targeting sequences of each gene (listed below) were annealed and ligated to the pLKO.1 cloning vector via AgeI and EcoRI sites. For lentiviral 160× GGGGCC repeat expression, the fragment of 80× GGGGCC was cut out from the pcDNA5-FRT-TO-(GGGGCC)_80_-NLuc by KpnI and SmaI, and cloned back via the KpnI and BssHII (blunt) sites upstream of the (GGGGCC)_80_ in the original pcDNA5-FRT-TO-(GGGGCC)_80_-NLuc plasmid in order to expand the repeat length to 160× GGGGCC repeats. Next, the fragment containing 160× GGGGCC repeat was cut out by KpnI and SmaI, and subcloned into the lentiviral vector under the EF1a promoter via KpnI and NheI (blunt). For the lentiviral plasmids expressing poly-GA or poly-GR with randomized codons, the sequences encoding the randomized 50×GA or 50×GR with ATG start codon were synthesized (Genewiz) and inserted in the lentiviral vector under the EF1a promoter with 2×FLAG at the 3’ end. The HiBiT tag was linked to the 5’ of APEX2 by PCR amplification from APEX2-OMM (Addgene #79056), and inserted upstream of GFP/GA50/GR50 at the XbaI and BamHI sites.

### Cell culture and transfection

Control and patient lymphoblast cells were maintained as described^23^ (Supplementary Table 1): cells were grown in RPMI1640 supplemented with 10% (v/v) FBS, 2mM L-Glutamine, 100 μg/ml streptomycin and 100 U/ml penicillin. HeLa Flp-In dual luciferase reporter cells^5,22,23^ and 293T cells were grown in DMEM supplemented with 10% (v/v) FBS, 100 μg/ml streptomycin and 100 U/ml penicillin. 293T cells were used for the lentivirus packaging. All cells were maintained at 37°C with 5% CO2.

Lipofectamine RNAiMAX (Thermo Fisher Scientific) was used to transfect siRNAs following the manufacturer’s protocol. The siRNAs targeting *METTL14* (Cat# M-005170-01), *ZCCHC8* (M-021026-00), and the Non-Targeting control were purchased from GE Dharmacon. Single siRNAs were used to knock down YTHDC1 (targeting sequence: 5'-GTCGACCAGAAGATTATGATA-3') and YTHDF2 (targeting sequence: 5′-AAGGACGTTCCCAATAGCCAA-3′) as previously described^9,15^. 293T cells were used to package lentivirus expressing non-targeting control shRNA (Addgene #10879), YTHDC1 shRNA (Sigma, #TRCN0000243989), ZCCHC8 shRNA (targeting sequence: 5'-TTAGCACTGAGAGCTATTTAA-3'), FTO shRNA (Sigma, #TRCN0000246247), METTL14 shRNA (targeting sequence: 5'-CCATGTACTTACAAGCCGATA-3'), and lentiviral METTL14 overexpression plasmid. Three biological replicates were prepared at each condition for RNA quantification and luciferase reporter experiments. The NLuc luciferase activity was measured by Nano-Glo Dual Luciferase Assay (Promega) on Tecan Infinite 200 PRO. NLuc levels were normalized to total protein amounts for each sample. Protein lysates were quantified by BCA Assay (ThermoFisher Scientific).

### Human iPSC and iPSC-derived neurons

C9ORF72-ALS/FTD and non-neurological control iPSC lines were obtained from the Answer ALS repository at Cedars-Sinai iPSC Core (see Supplementary Table 2 for demographics). Presence or absence of the C9ORF72 repeat expansion was verified by repeat-primed PCR (RP-PCR)^56^. iPSCs and iPS-neurons were routinely checked for the expression of poly(GP) level. iPSCs were maintained in mTeSR media on growth factor reduced Matrigel (Corning, Cat# 354230), following the Cedars-Sinai Standard Operating Procedure (SOP, https://www.cedars-sinai.edu/content/dam/cedars-sinai/research/documents/biomanufacturing/complete-ipsc-culturing-protocol-rev-4-2020.pdf). iPSCs were differentiated into spinal neurons using the direct induced motor neuron protocol as described^5^. The i^3^Neuron iPSC line^19^ was obtained from Dr. Michael Ward’s lab. The i^3^Neuron iPSCs were cultured in Essential 8 media (Thermo Fisher Scientific) on Matrigel (Corning, Cat# 354277), differentiated into i^3^Neuron using the protocol as described^19^, and harvested on Differentiation Day 14. All cells were maintained at 37°C with 5% CO2.

To overexpress METTL14 or knock down FTO in C9ORF72-ALS/FTD iPSNs, neurons were transduced with lentivirus expressing GFP or METTL14, or control shRNA or FTO shRNA on Day 18 post-differentiation, and then harvested 7 days after infection for protein and RNA assays, or maintained till Day-32 differentiation for glutamate-induced excitotoxicity assay. The FTO inhibitor CS2 (also named brequinar, Cayman Chemical, Cat# 24445) was added at 500nM on Day 16 post-differentiation, and the neurons were harvested after 7 days for protein and RNA assays. For glutamate-induced excitotoxicity assay, CS2 (500nM) was added on differentiation day 25 and the assay was performed on day 32. Neurons were fed with fresh media with CS2 every other day. To harvest relatively pure neuron population for protein and RNA assays from the iPSN culture, iPSNs were gently washed once with 1×PBS, and then collected by using 1×PBS to wash off the neuron matrix followed by bench-top spin and 1×PBS wash.

For glutamate-induced excitotoxicity of patient iPSNs, neurons were treated with 0 or 10μM L-glutamate for 4 hours on day 32 of differentiation. After 3.5 hours, the cells were stained with Hoechst33342 (5μg/ml) and Propidium Iodide (1μg/ml) for 30 min to visualize total and dead cells under ZEISS Axio Observer^57,58^. Images were taken randomly. At least 350 neurons were quantified per replicate blindly.

For expressing 160× GGGGCC repeat or poly-GA/GR with randomized codons using i^3^Neurons, cells were transduced with lentivirus expressing the different transgenes on differentiation day 5 at an MOI of 1.5 in Cortical Neuron Culture Medium^19^, and harvested on differentiation day 14 for the measurement of endogenous *METTL3* and *METTL14* expression. The expression of poly-DPR constructs post transduction was measured via the Nano-Glo HiBiT Lytic Detection System (Promega, Cat# N3030), and visualized by immunofluorescent with the FLAG antibody (Sigma, F1804, 1:500).

For luciferase assays using i^3^Neurons, cells were first transduced with lentivirus expressing luciferase reporters on differentiation day 2 at an MOI of 1 in Cortical Neuron Culture Medium^19^. On differentiation day 5, the cells were transduced with lentivirus expressing non-targeting control shRNA, METTL14 shRNA, YTHDC1 shRNA or ZCCHC8 shRNA. Semi-media change was performed every other day till the harvest day. i^3^Neurons were harvested on differentiation day 14 for luciferase and RNA assays.

For all assays, the cells were plated randomly in multiple-well plates and were randomly assigned to different experiment groups.

### Human tissue and immunohistochemistry

C9ORF72-ALS/FTD patient and non-neurological control postmortem brain tissues and paraffin-embedded temporal cortex sections were obtained from Johns Hopkins ALS Postmortem Tissue Core and Target ALS Postmortem Tissue Core with approval from patient consent (see Supplementary Table 3 and 4 for demographic information). The study using patient samples/data was approved by the Johns Hopkins University School of Medicine Office of Human Subjects Research Institutional Review Boards. Tissue sections were gradually rehydrated with xylene twice for 10 min, 100% ethanol twice for 10 min, 95% ethanol twice for 5 min, 70% ethanol for 5 min, 50% ethanol for 5 min, 30% ethanol for 5 min, PBS for 5 min and finally dH2O for 5 min. Antigen retrieval was performed with 0.1M sodium citrate buffer (pH 6.0) for 5min in a steam sterilizer. The slides were permeabilized with 0.2% Triton X-100 for 20min and blocked with the blocking buffer (1% BSA and 2% Goat Serum diluted in 1× PBS) for 1 hour. Slides were incubated in each primary antibody diluted in the blocking buffer overnight at 4°C. On the next day, slides were washed three times for 5 min with 1× PBS, and treated with 0.3% H_2_O_2_ for 30min. After incubation with secondary antibody (Goat Anti-Rabbit IgG (H+L), Biotinylated, Vector Laboratories BA-1000-1.5, 1:300) for 1 hour, the slides were treated with the R.T.U. VECTASTAIN Elite ABC Reagent for 30min. Finally, the slides were developed using the DAB Peroxidase Substrate Kit (Vector Laboratories SK-4100) for exactly 1 minute each. Slides stained with METTL14 were also counterstained before mounting. Slides were imaged with Keyence BZ-X all-in-one fluorescent microscope. All images were acquired using identical exposure time and conditions. Primary antibodies: METTL14 (Sigma-Aldrich HPA038002, 1:500), METTL3 (Abcam ab195352, 1:500), YTHDC1 (Sigma-Aldrich HPA036462, 1:500), YTHDF2 (Abcam ab246514, 1:2500).

### Immunoblotting

Samples were homogenized in RIPA buffer supplemented with 1× cOmplete protease inhibitor cocktail (Sigma). Homogenates were spun at 21,130g for 10 min at 4°C. The protein concentrations of supernatants were determined using BCA protein assay (Thermo Fisher Scientific 23224). Equal amount of protein samples was loaded on 10% acrylamide gel and transferred to nitrocellulose membrane (GE Healthcare). Membrane was blocked in 5% non-fat milk/TBST for 30-60 min at room temperature and incubated with primary antibody (1:1000) overnight at 4 °C. After TBST wash, the membrane was incubated with HRP-linked secondary antibody (Cytiva, 1:10,000) for 1 hour. Protein bands were detected using Clarity Western ECL Substrate kit (Bio-Rad) or SuperSignal West Pico PLUS Chemiluminescent Substrate kit (Thermo Fisher Scientific) and imaged using a ChemiDoc Imager (Bio-Rad). Analysis was conducted using Fiji. Inter-group differences were assessed by two-tailed Student's *t*-test. Primary antibodies: METTL14 (Sigma-Aldrich HPA038002), METTL3 (Abcam ab195352), YTHDC1 (Sigma-Aldrich HPA036462), YTHDF2 (Abcam ab220163) and ZCCHC8 (Proteintech 23374-1-AP).

### RNA isolation and RT-qPCR

Total RNAs were isolated with TRIZOL reagent (Invitrogen). RNA samples used for RT-qPCR were treated with RQ1 DNase I (Promega) and then used for complementary DNA (cDNA) synthesis with random hexamers (Thermo Fisher Scientific). qPCR was performed using SYBR Green Master Mix (Thermo Fisher Scientific) on the CFX96 real-time PCR detection system (Bio-Rad). The *C9ORF72* repeat-containing intron RNA levels in patient samples were measured using TaqMan fast advanced master mix (Thermo Fisher Scientific). For the quantification of antisense repeat RNA levels^31^, the reverse transcription was performed using the antisense strand-specific primer (Supplementary Table 5) via the Superscript III reverse transcriptase (Thermo Fisher Scientific), and then subjected to qPCR using TaqMan fast advanced master mix. Custom primer sequences are listed in Supplementary Table 5. Human ACTB primer set (Hs.PT.56a.40703009.g) was purchased from Integrated DNA technologies. At least three biological replicates with each containing two technical replicates were used for quantification. *GAPDH* or *HPRT1* mRNA was used as the internal control. Inter-group differences were assessed by two-tailed Student's *t*-test.

### LC-MS/MS quantification of m^6^A in RNA

Non-ribosomal RNA was extracted from the total RNA using the RiboMinus transcriptome isolation kit (Thermo Fisher Scientific) following the manufacturer’s protocols. Polyadenylated RNA (poly-A RNA) was purified using NEBNext Poly(A) mRNA Magnetic Isolation Module (New England Biolabs). 50 ng of poly-A RNA or non-ribosomal RNA was digested with 1U nuclease P1 in 30μl of buffer containing 20 mM of NH_4_OAc for 2 h at 42 °C. 1× FastAP Buffer and 1U FastAP Thermosensitive Alkaline Phosphatase (Life Technology) were added to the samples. The reaction was carried out at 37 °C for an additional 2 hours, and then centrifuged through filters (0.22 μm pore size, 4 mm diameter, Millipore). Nucleosides in the filtered samples were separated by reverse phase ultra-performance liquid chromatography (C18 column) and detected by Triple Quad 6500 System (AB SCIEX) in a positive ionization mode. The nucleosides were quantified by using the nucleoside-to-base ion mass transitions of 282 to 150 (m^6^A), and 268 to 136 (A). A standard curve was obtained from pure nucleoside standards in the same batch of samples. The m^6^A/A ratio was calculated based on the calibrated concentrations comparing to the standard curve.

### Immunoprecipitation

One 10cm dish of neurons per line were harvested on day 18 of differentiation and washed two times with cold PBS. After centrifugation at 211g for 5min, the neuron pallet was lysed in 400μl lysis buffer, composed of 20mM Tris pH7.4, 200mM NaCl, 0.5% NP-40, 1mM DTT, 0.1mM EDTA, 0.1mM EGTA, 1× cOmplete protease inhibitor cocktail (Sigma) and SUPERase-in RNase Inhibitor (0.25μl/100μl, Thermo Fisher Scientific). The lysate was sheared with 26-gauge needle for 6 times, incubated on ice for 5min, and subsequently centrifuged at 21,130g for 20min at 4. Protein G Dynabeads were washed once with PBST (PBS with 0.02% Tween-20) and incubated with YTHDC1 antibody (Sigma-Aldrich HPA036462) or rabbit IgG (Sigma) four-volume of wash buffer with rotation for 1 hour at RT. The antibody-coated beads were washed three times with lysis buffer and incubated with the supernatant of the cell lysates at 4 °C for 1 hour with rotation. After the incubation, the beads were wash five times with lysis buffer. The proteins bound on the beads were eluted in SDS loading buffer and heated at 95°C for 5min.

### MeRIP-seq and MeRIP-RT-qPCR

Ribosomal RNA was depleted from total RNA by RiboMinus Eukaryote Kit v2 (Thermo Fisher Scientific). m^6^A-immunoprecipitation (m^6^A-IP) was performed using the EpiMark N6-Methyladenosine Enrichment Kit (New England Biolabs) following the manufacturer’s protocols. Library preparation was performed using the SMARTer Stranded Total RNA-Seq Kit v2 (Takara). Sequencing was performed at the University of Chicago Genomics Facility on an Illumina NovaSeq 6000 system for paired-end 50bp. For MeRIP-RT-qPCR, the m^6^A-modified *GLuc* RNA (NEB) was spiked in as the reference control before the m^6^A-immunoprecipitation. RNAs were extracted from m^6^A -IP and input fractions, and subjected to reverse transcription and qPCR. The *GLuc* RNA was used as normalization control. The fold enrichment of m^6^A-IP was compared to the input. Inter-group differences were assessed by two-tailed Student’s *t*-test. Rabbit IgG antibody was used as the negative control.

### YTHDF2 RNA immunoprecipitation (RIP)-seq

The iPSNs were harvested on day 22 of differentiation. The cell pallets were resuspended in equal volume of RIP lysis buffer (50mM HEPES, 150mM KCl, 2mM EDTA, 0.5% NP40, 0.5mM DTT, supplemented with 1× cOmplete protease inhibitor cocktail (Sigma), 0.25μl/100μl SUPERase-in RNase Inhibitor (Thermo Fisher Scientific)). The lysates were sheared with 26-gauge needle for 6 times, lysed on ice for 5min and centrifuged at 14,000g for 30min at 4 °C. Protein A/G beads (Thermo Fisher Scientific) were washed once with RIP lysis buffer, and incubated with YTHDF2 (Abcam ab246514) or rabbit IgG antibody (Sigma) for 1 hour at 4 °C. The antibody-coated beads were washed two times with RIP lysis buffer. The supernatants of the lysates were added to the pre-cleared beads and rotate at 4 °C for 4 hours. 5% of the lysates was saved as input and mixed with 1ml TRIZOL for RNA extraction. The beads were washed 6 times with NT2 buffer (50mM HEPES, 200mM NaCl, 2mM EDTA, 0.05% NP40, 0.5mM DTT, protease inhibitor and RNase inhibitor). After washing, 1ml TRIZOL was added to the beads to elute the RNA. RNAs recovered from RIP and input were subjected to NEBNext Ultra II Directional RNA Library Prep (New England Biolabs) coupled with NEBNext Poly(A) mRNA Magnetic Isolation Module (New England Biolabs).

### Meso Scale Discovery (MSD) ELISA

The endogenous GP level from iPSNs was quantified as previously described^5^. Briefly, the streptavidin coated plates were blocked in 1% blocker A in PBS (all reagents from MSD) overnight at 4 °C and coated with 0.375 μg/mL biotinylated rabbit anti-GP antibody. 35 μl of cell lysates was added in duplicate, along with a standard curve of GP8 peptide. Following a 3-hour incubation at room temperature, wells were washed with PBST, and sulfo-tagged detection antibody (0.5 μg/mL) was added. After incubation and washes, read buffer was added followed by immediate imaging using MESO QuickPlex SQ 120.

### RNA fluorescent in situ hybridization (FISH)

LNA-incorporated DNA probes with TYE-488 dye (Exiqon) were used against the (GGGGCC)n RNA repeats or (CCCCGG)n RNA repeats^27^. Cells growing on coated coverslips were fixed with 4% PFA at room temperature for 15min and permeabilized with 0.25% Triton X-100 in PBS for 15 min. The cells were incubated with the pre-hybridization buffer (10% Dextran Sulfate, 10% deionized formamide in 2× SSC supplemented with 0.1mg/mL E.coli tRNA, 10mM Ribonucleoside Vanadyl Complex (NEB #S1402S), and SUPERaseIn RNase inhibitor (Ambion)) at room temperature for 30 min. The FISH probes for sense repeats and antisense repeats were diluted to 50 nM in the hybridization buffer and incubated with the cells at 60 °C for 3 hours, followed by two washes with 10% formamide in 2× SSC at 60 °C for 10 min each and three washes with 2× SSC at room temperature. Cells were then stained with DAPI and mounted for imaging. FIJI was used to quantify RNA foci.

### Proteomic data analysis

Proteomic data matrix was downloaded from NeuroLINC and Answer ALS databases (see Supplementary Table 6 for demographics). Proteins with absent values in more than 4 samples were removed from the list. For Answer ALS data, protein expression among each batch was normalized using the value of the corresponding AE8 iPSN batch control. Z-score was calculated for each protein between the control and patient samples of NeuroLINC or AnswerALS. Wilcox test was used to determine significance.

### RNA decay measurement

5 μg/ml Actinomycin D was added to HeLa Flp-In dual luciferase reporter cells or day-32 iPSNs at 6 h, 3 h, and 0 h before collection for RNA extraction by Trizol. For RNA-seq, same proportion of ERCC RNA spike-in control (Ambion) was added to each sample before poly-A selection. Library preparation was performed using NEBNext Ultra II Directional RNA Library Prep (New England Biolabs) coupled with NEBNext Poly(A) mRNA Magnetic Isolation Module (New England Biolabs). To measure the half-life of *C9ORF72* repeat RNA, equal amount of *CLuc* RNA (New England Biolabs) was spiked in before reverse transcription as normalization control for qPCR. Sequencing was performed on a NovaSeq platform for paired-end 150bp.

### m^6^A-seq analysis

Raw reads were trimmed with Trimmomatic-0.39^59^, then aligned to human genome and transcriptome (hg38) using HISAT (version 2.1.0)^60^ with ‘--rna-strandness RF’ parameters. Annotation files (version v29, 2018-08-30, in gtf format) were downloaded from GENCODE database (https://www.gencodegenes.org/). Mapped reads were separated by strands with samtools (version 1.9)^61^ and m^6^A peaks on each strand were called using MACS (version 2)^62^ with parameter ‘-nomodel, --keep-dup 5, -g 2.052e9, --tsize 60 -extsize 150’ separately. Peaks with q < 0.01 were considered as significant ones. Significant peaks identified in at least three biological replicates were merged using bedtools (v.2.26.0)^61^ and were used in the subsequent analysis. Differentially methylated peaks were called with MeTDiff^63^ with default parameters.

### RNA-seq analysis

Raw reads were trimmed with Trimmomatic-0.39^59^, then aligned to human genome and transcriptome (hg38) using HISAT (version 2.1.0)^60^ with ‘--rna-strandness RF’ parameters. Annotation files (version v29, 2018-08-30, in gtf format) were downloaded from GENCODE database (https://www.gencodegenes.org/). Reads on each GENCODE annotated gene were counted using HTSeq^64^ and then differentially expressed genes were called using DESeq2 package in R^65^ requiring at least 10 read counts in at least three samples with p value < 0.05.

### RNA half-life analysis

Raw reads were trimmed with Trimmomatic-0.39^59^, then aligned to human genome and transcriptome (hg38) and external RNA Control Consortium (ERCC) RNA spike-in control (Thermo Fisher Scientific) using HISAT (version 2.1.0)^60^ with ‘--rna-strandness RF’ parameters. Annotation files (version v29, 2018-08-30, in gtf format) were downloaded from GENCODE database (https://www.gencodegenes.org/). Reads on each GENCODE annotated gene were counted using HTSeq^64^ and then normalized to counts per million (CPM) using edgeR^66^ packages in R. CPM was converted to attomole by linear fitting of the RNA ERCC spike-in. Half-life of RNA was estimated using formula listed in the previous publication^24^. Specifically, as actinomycin D treatment results in transcription stalling, the change of RNA concentration at a given time (dC∕dt) is proportional to the constant of RNA decay ($K_{decay}$) and the RNA concentration (C), leading to the following equation:

$$\frac{dC}{dt}=-K_{decay}C$$

Thus, the RNA degradation rate $K_{\text{decay}}$ was estimated by:

$$\text{ln}\left(\frac{C}{C_{0}}\right)=-K_{decay}t$$

To calculate the RNA half-life ($t_{1∕2}$), when 50% of the RNA is decayed (that is, $\frac{C}{C_{0}}=\frac{1}{2}$), the equation was:

$$\text{ln}\left(\frac{1}{2}\right)=-K_{decay}t_{\frac{1}{2}}$$

From where:

$$t_{\frac{1}{2}}=\frac{ln2}{K_{decay}}$$

The final half-life was calculated by using the average value of 0 h, 3 h and 6 h.

### YTHDF2 RIP-seq analysis

Raw reads were trimmed with Trimmomatic-0.39^59^, then aligned to human genome and transcriptome (hg38) using HISAT (version 2.1.0)^60^ with ‘--rna-strandness RF’ parameters. Annotation files (version v29, 2018-08-30, in gtf format) were downloaded from GENCODE database (https://www.gencodegenes.org/). Reads on each GENCODE annotated gene were counted using HTSeq^64^ and then differentially expressed genes were called using DESeq2 package in R^65^. Differentially upregulated genes requiring at least 10 read counts in at least three samples with p adjusted < 0.05 were identified as YTHDF2 target genes.

### Enrichment analysis

Motif enrichment analysis was performed with HOMER (version 4.11)^67^ in RNA mode, and GO enrichment analysis was performed with DAVID (version 6.8)^68-70^ with default parameter.

### Statistics and reproducibility

Data shown were from at least three independent biological replicates or individual control or patient cell lines/tissue samples. Quantitative data were presented as mean with standard error of the mean (SEM). R or Prism (GraphPad Software) were used for statistical analysis, including Student’s t-test, one-way ANOVA with multiple testing, non-parametric Wilcoxon-Matt-Whitney test, Fisher’s exact test where appropriate. Statistical significance defined as p<0.05 (∗), p<0.01 (∗∗), p<0.001 (∗∗∗), and p < 0.0001 (∗∗∗∗). Statistical details of experiments can be found in the figure legends. No statistical methods were used to pre-determine sample sizes but our sample sizes are similar to those reported in previous publications^5,22,23,24^.

## Extended Data

## Supplementary Material

Supplementary Tables

## Data Availability

Requests for further information or resources and reagents should be directed to and will be filled by the lead contact, Shuying Sun (shuying.sun@jhmi.edu). Plasmids generated in this study are available from the lead contact upon request. The sequencing data (Supplementary Table 7) have been deposited at the NCBI under GEO accession number GSE203581.

## Code availability

This paper does not report original code.
